# Fit‐for‐purpose heterodivalent single‐domain antibody for gastrointestinal targeting of toxin B from *Clostridium difficile*

**DOI:** 10.1002/pro.5035

**Published:** 2024-06-26

**Authors:** Everardo R. Rodriguez Rodriguez, Rune Thorbjørn Nordvang, Marcus Petersson, Jakob Kræmmer Haar Rendsvig, Emma Wenzel Arendrup, Monica L. Fernández Quintero, Timothy P. Jenkins, Andreas H. Laustsen, Sandra Wingaard Thrane

**Affiliations:** ^1^ Bactolife A/S Copenhagen East Denmark; ^2^ Department of Biotechnology and Biomedicine Technical University of Denmark Lyngby Denmark

**Keywords:** antibody, bile salt stability, *Clostridium difficile*, developability, oral administration, single‐domain antibody

## Abstract

Single‐domain antibodies (sdAbs), such as V_H_Hs, are increasingly being developed for gastrointestinal (GI) applications against pathogens to strengthen gut health. However, what constitutes a suitable developability profile for applying these proteins in a gastrointestinal setting remains poorly explored. Here, we describe an in vitro methodology for the identification of sdAb derivatives, more specifically divalent V_H_H constructs, that display extraordinary developability properties for oral delivery and functionality in the GI environment. We showcase this by developing a heterodivalent V_H_H construct that cross‐inhibits the toxic activity of the glycosyltransferase domains (GTDs) from three different toxinotypes of cytotoxin B (TcdB) from lineages of *Clostridium difficile*. We show that the V_H_H construct possesses high stability and binding activity under gastric conditions, in the presence of bile salts, and at high temperatures. We suggest that the incorporation of early developability assessment could significantly aid in the efficient discovery of V_H_Hs and related constructs fit for oral delivery and GI applications.

## 1 INTRODUCTION

Monoclonal antibodies and antibody fragments have emerged as the largest class of molecules in clinical development, offering reliable solutions for diagnosing and treating a wide array of diseases (Walsh & Walsh, 2022). However, their applicability extends beyond traditional medicine, highlighting their versatility in addressing various health challenges (Gavrilaș et al., 2022; Harris, 1999; Hortigüela & Wall, 2013; Petersson et al., 2023).

A critical aspect common to all antibody‐based applications is their developability. The identification of developable antibodies involves the evaluation of functional and physicochemical properties, as well as ease of recombinant expression, to assess if the antibodies are likely to transition well from discovery to the intended applications (Fernández‐Quintero et al., 2023). While established methods for assessing antibody developability in therapeutic applications exist (Ausserwöger et al., 2022; Bailly et al., 2020; Wolf Pérez et al., 2022), it is less explored what constitutes a good developability profile outside the realm of injectable therapeutics. The gap is particularly evident in the emerging area of single‐domain antibodies (sdAbs), or V_H_Hs, especially in their application for reducing the risk of contracting gastrointestinal (GI) infections through oral delivery (Debatis et al., 2023; Fiil et al., 2022; Petersson et al., 2023). The ability of V_H_Hs to survive passage through the GI tract and effectively bind to their targets at the correct time, anatomical location, and epitope is crucial. This ensures that their mode of action is aligned with their intended purpose, a consideration that is particularly important for targeting GI infections (Debatis et al., 2023; Pitiot et al., 2022; Reilly et al., 1997; Tsubokura et al., 2003). Thus, the intricacies in developing antibodies for oral administration highlight the need for innovative approaches, and efficient experimental methods, to aid selection of the most developable molecules.

Some categories of antibody targets may require special attention during the antibody development process. For instance, neutralization of protein‐based bacterial toxins, which were among the first targets used as antigens in antibody research, can often be achieved by directly binding to a neutralizing epitope (Drozdowski et al., 2010; Garcia‐Rodriguez et al., 2018; Rouha et al., 2018), as exemplified by the very first antisera containing polyclonal antibodies (Behring, 1890). However, neutralization of specific bacterial toxins can be challenging due to their intricate biochemistry, biological behavior, and/or unique pharmacology (Carter et al., 2015; do Vale et al., 2016; Ghazaei, 2022). Binding toxins with antibodies may even lead to undesirable effects such as antibody‐dependent enhancement of toxicity (ADET) (Faulstich et al., 1988; Morens, 1994; Sánchez‐Zuno et al., 2021; Sørensen et al., 2023; Torres et al., 2021), a phenomenon that has been observed for toxin A (TcdA) from *Clostridium difficile* (He et al., 2009). These aspects are thus of high importance when assessing the developability of antibody‐based molecules early in the discovery process, ensuring their functionality and stability under application‐relevant conditions.

**FIGURE 1:**
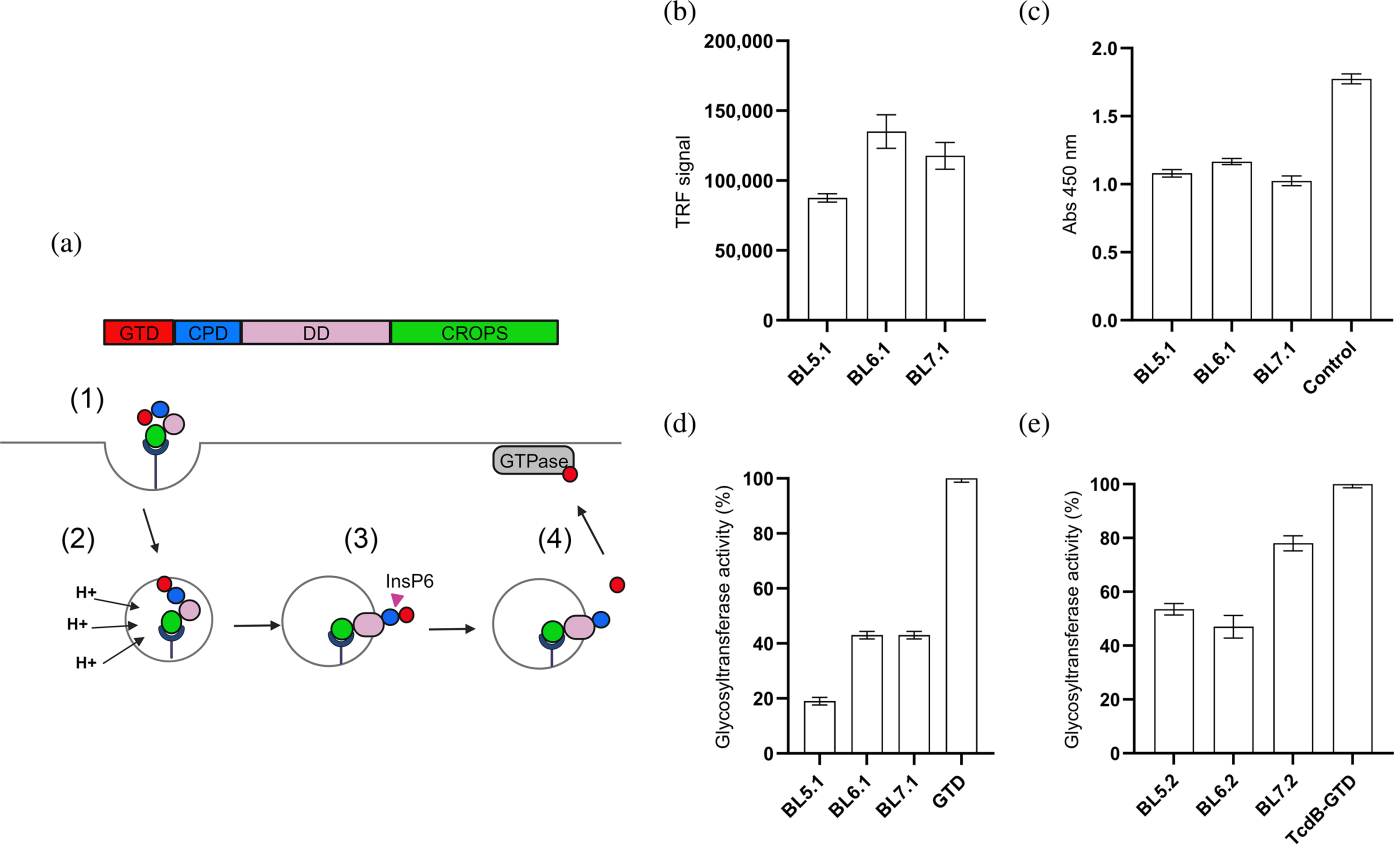
Assessment of the binding and blocking capacity of selected monovalent and homodivalent V_H_H constructs towards recombinant and native GTD from TcdB. (a) Schematic representation of the glucosyltransferase GTPase‐dependent mechanism of action of TcdB, highlighting the process of receptor‐mediated internalization (1), pH‐induced conformational changes (2), translocation, and release of GTD into the cytosol via InsP6‐dependent autoproteolysis (3). Once in the cytosol, the active GTD glycosylates GTPases present in the cell (4), causing cytoskeletal disruption and cell dysregulations, which ultimately leads to cell death. CPD, cysteine protease domain; CROPS, combined repetitive oligopeptides domain; DD, delivery domain; GTD, glucosyltransferase domain. (b) Binding assessment of three monomeric V_H_Hs against recombinant GTD. (c) Binding assessment of monomeric V_H_Hs to native TcdB using ELISA, compared to the control immunoglobulin G (IgG) antibody, bezlotoxumab. The graph shows binding signal for each binder in absorbance units. (d) In vitro evaluation of the capacity of the monomeric V_H_Hs to inhibit recombinant GTD enzyme activity. GTD activity (%) was calculated by comparing glycosyltransferase activity in the presence or absence of individual V_H_Hs at a molar ratio of 1:20 (GTD:V_H_H). (e) Assessment of the inhibitory capacity of homodivalent V_H_H constructs against the native GTD after in vitro induction of GTD cleavage. GTD activity (%) was calculated by comparing glycosyltransferase activity in the presence or absence of individual homodivalent V_H_H constructs at a molar ratio of 1:20 (GTD:‘V_H_H binding sites’, i.e., 1:10 GTD:‘homodivalent V_H_H’). The graphs (b‐e) represent the mean of two independent experiments, each performed in duplicates with standard deviation (SD) for each group plotted as error bars.

Here, we explored the developability properties of homo‐ and heterodivalent V_H_H constructs against toxin B (TcdB) from *C. difficile*; a critical virulence factor characterized by four functional domains (Aktories et al., 2017; Pruitt et al., 2010). Divalent V_H_H constructs have previously been explored for GI applications due to their intrinsic high stability and increased avidity compared to monovalent formats (Debatis et al., 2023; Fiil et al., 2022; Kang & Seong, 2020; Petersson et al., 2023). The mechanism of action of TcdB involves receptor‐mediated internalization and conformational changes under acidic conditions, leading to the translocation of the glycosyltransferase domain (GTD) of the toxin into the cytosol, where it causes cytoskeletal disruption and cell death upon release via auto‐cleavage (Figure 1a) (Carter et al., 2015; Chen et al., 2019; Orrell et al., 2017). To optimize the chances of identifying V_H_H constructs that function under the conditions where TcdB is produced and active, we assessed the binding efficiency of such V_H_Hs taking both the conformational changes of TcdB and GI conditions into consideration. These conditions included different pH levels, presence of proteases, and both primary and secondary bile salts. The latter are crucial, as they induce conformational shifts in TcdB towards a more compact state (Tam et al., 2020). We also assessed the stability of the V_H_H constructs at the different temperatures that they may be subjected to during large‐scale bio‐industrial manufacture and processing, which is needed for products that are ingested (e.g., pasteurization).

Finally, to include functionality in the developability assessment of our selected fit‐for‐purpose heterodivalent V_H_H construct, we compared its ability to inhibit the toxic effects of TcdB in a mammalian colon‐derived cell line to a control V_H_H construct known to neutralize TcdB in vitro and in vivo (Yang et al., 2014).

## 2 RESULTS

### 2.1 Camelid V_H_H libraries and phage display panning

From two alpacas immunized with GTD, individual V_H_H‐displaying phage libraries were generated, with sizes of 9.5 × 10^8^ transformants (89% insert rate) and 1 × 10^9^ transformants (91% insert rate). Initially, each library was individually panned against recombinant GTD. The outputs were combined for the second and third panning round, achieving enrichments of 1,700‐fold and 8,000‐fold, respectively. Afterwards, 376 monoclonal V_H_Hs from individually picked colonies were expressed in 96‐well plates, and the supernatants were used for screening their ability to bind to GTD.

### 2.2 Binding and inhibition of the glycosyltransferase domain of toxin B

The top seven V_H_Hs with the highest binding strength to GTD, measured by time‐resolved fluorescence (TRF) using normalized dissociation‐enhanced lanthanide fluorescence immunoassay (DELFIA), were sequenced, and the amino acid sequences were compared and clustered into three groups based on their CDR3 sequences. We selected three unique binders, representative of each of the three clusters, that showed the highest binding signal for further study: BL5.1, BL6.1, and BL7.1. The BL6.1 exhibited the highest TRF signal (147,000 units), followed by BL7.1 (108,000 units), and BL5.1 (85,000 units) (Figure 1b). Their binding to native GTD from TcdB was confirmed via whole‐toxin enzyme‐linked immunosorbent assay (ELISA) (Figure 1c), aligning with initial rankings, with BL6.1 binding the strongest (Abs: 1.1), followed by BL5.1 (Abs: 1.0), and then BL7.1 (Abs: 0.9). While binding indicates specificity, functionality is key. Therefore, we screened the V_H_Hs for their ability to inhibit the enzymatic activity of the recombinant GTD in vitro. Here, BL5.1 most effectively reduced GTD activity by 80%, compared to 55% for BL6.1 and BL7.1 (Figure 1d).

### 2.3 Homodivalent V_H_H constructs inhibit the activity of native GTD

Homodivalent V_H_H constructs (BL5.2, BL6.2, and BL7.2) were generated by fusing two monovalent V_H_Hs with a Gly‐Ser linker (GGGGS)_3_, and the ability of these constructs to inhibit the native GTD from TcdB was assessed in vitro. This involved mimicking the mechanism of action of TcdB at the stage where GTD is released from the holotoxin (Figure 1a), prior to measuring its activity. In these experiments, it was shown that the enzymatic activity of native GTD, activated through auto‐cleavage in vitro (verified by SDS‐PAGE, see Figure S1), could be reduced with approximately 20% by BL7.2 and around 50% by BL5.2 and BL6.2 (Figure 1e).

### 2.4 Kinetic parameters driving the V_H_H–GTD interaction

Using bio‐layer interferometry (BLI) and surface plasmon resonance (SPR), we determined the affinity (*K*_D_) and apparent affinity (*K*_Dapp_) for the monovalent and homodivalent V_H_H constructs, respectively, to the recombinant GTD, as well as the mean residence time (*T*_R_). All the V_H_H constructs displayed affinities in the sub‐ or low nanomolar range (0.01–10 nM), indicating strong binding interactions with GTD (Table 1). The monovalent BL5.1 and BL6.1 exhibited slower *k*_off_ values compared to BL7.1, with mean residence times of 5.5 and 2 h, respectively. Meanwhile, all the homodivalent V_H_H constructs demonstrated improved apparent affinity, in the sub‐nanomolar range, compared with their monomeric versions, suggesting the presence of avidity effects (Table 1).

**TABLE 1:** Kinetic parameters for the V_H_H–GTD binding interactions.

V_H_H	*k*_on_ (M^−1^s^−1^)	*k*_off_ (s^−1^)	*K*_D_/*K*_Dapp_	Chi^2^	*R* ^2^	*T*_R_ (min)
BL5.1	4.60 × 10^4^ (±1.94 × 10^1^)	5.03 × 10^−5^ (±2.03 × 10^−7^)	1.09 × 10^−9^ (±5.1 × 10^−8^)	1.49 × 10^−1^		333
BL6.1	2.87 × 10^5^ (±3.2 × 10^2^)	1.44 × 10^−4^ (±5.2 × 10^−7^)	5 × 10^−10^ (±1.9 × 10^−12^)		0.9984	116
BL7.1	2.03 × 10^6^ (±1.4 × 10^4^)	2.07 × 10^−2^ (±4.3 × 10^−5^)	1.01 × 10^−8^ (±7.4 × 10^−11^)		0.9933	0.02
BL5.2	2.05 × 10^4^ (±3.89 × 10^1^)	1.91 × 10^−9^ ^a^ (±3.12 × 10^−9^)	9.3 × 10^−14^ ^b^ (±7.4 × 10^−12^)	1.39		<1.6 × 10^5^
BL6.2	7 × 10^5^ (±1.7 × 10^3^)	<1 × 10^−7^ ^a^ (±<1 × 10^−7^)	<1 × 10^−12^ ^b^ (±7.4 × 10^−12^)		0.9936	<1.6 × 10^5^
BL7.2	2.2 × 10^3^ (±2.66 × 10^2^)	<1 × 10^−7^ ^a^ (±<1 × 10^−7^)	2.14 × 10^−11^ ^b^ (±1 × 10^−10^)		0.9927	<1.6 × 10^5^

*Note*: Kinetic parameters for the selected monovalent and homodivalent V_H_H constructs. The measurements of the association rate constant (*k*_on_) and the dissociation rate constant (*k*_off_) are used to calculate equilibrium dissociation constant (*K*_D_), the coefficient of determination (*R* ^2^), Chi^2^, and mean time of residence (*T*_R_). Values obtained were calculated using a 1:1 global fitting model including ±standard deviation. *T*_R_ was calculated using *k*_off_ values translated to minutes.

^a^ *k*_off_ values below the limit of detection of the equipment.

^b^ Apparent affinity values (*K*_Dapp_) determined for the homodivalent V_H_H constructs.

### 2.5 Monovalent and homodivalent V_H_H constructs retain functionality under simulated gastric conditions and thermal stress

**FIGURE 2:**
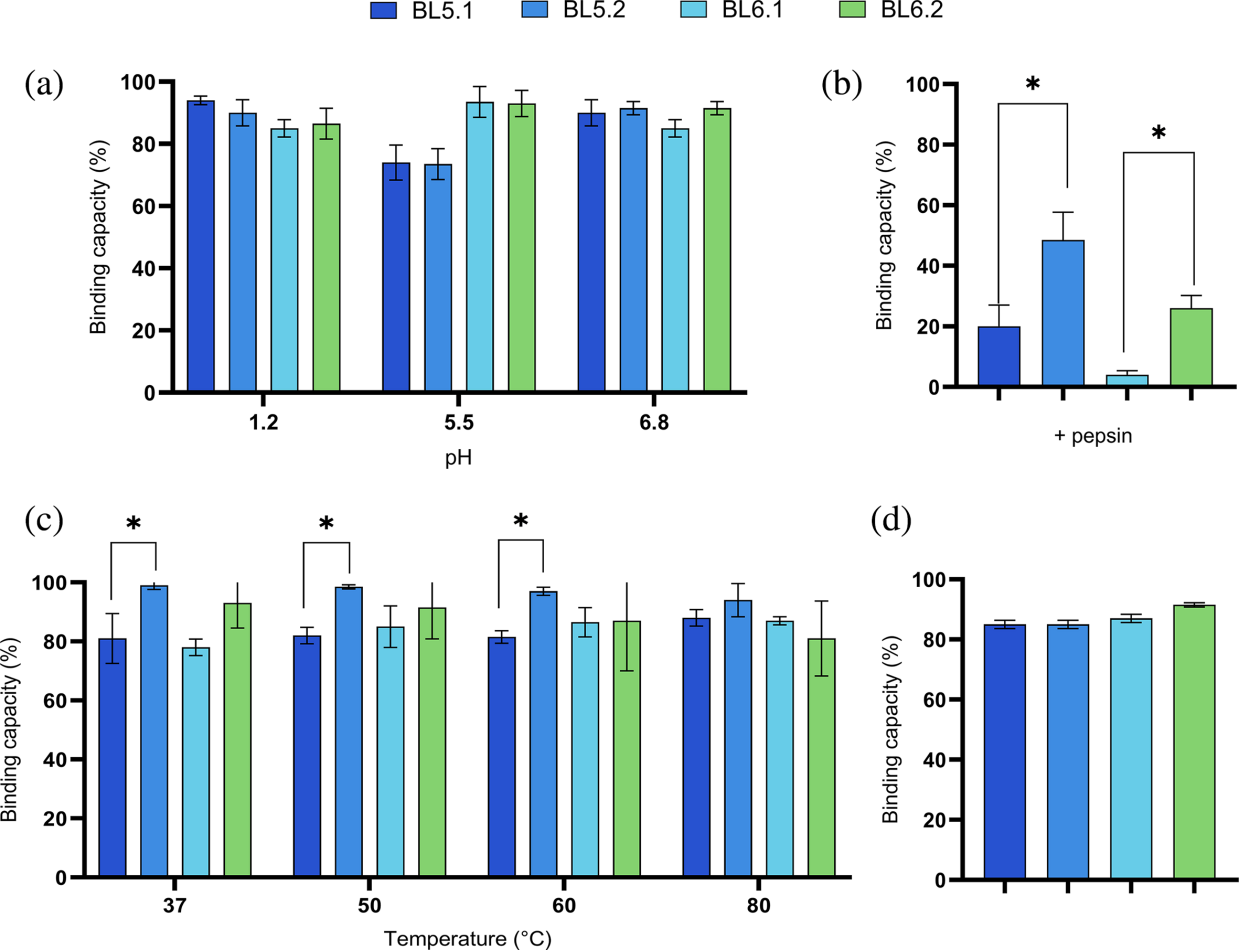
Assessment of the functional stability of monovalent and homodivalent V_H_H constructs. (a) Assessment of stability at physiologically relevant pH (pH 1.2, pH 5.5, and 6.8), according to Maffey et al. (2016). The graph shows the retained binding capacity (%) of the homodivalent V_H_H against GTD after pH exposure. (b) Retained binding capacity of monovalent and homodivalent V_H_H constructs after exposure to pepsin (1 mg/mL). (c) Assessment of thermostability. The graph shows retained binding capacity (%) of the monovalent and homodivalent V_H_H constructs after incubation at temperatures ranging from 37 to 80°C for 1 h. (d) Assessment of food product compatibility and shelf‐life stability. The graph shows retained binding capacity (%) of the monovalent and homodivalent V_H_H constructs after incubation with skimmed milk for 7 days at 4°C following an high‐temperature short‐time (HTST) pasteurization. For all graphs (a‐d), binding capacity of untreated homodivalent V_H_H samples was set to 100% binding capacity and the remaining binding capacity of treated samples was calculated as the binding capacity relative thereto. Furthermore, all graphs (a–d) represent the mean of two independent experiments, each in duplicates with SD for each group indicated by error bars. Statistical difference was calculated using the unpaired Mann–Whitney test (*p < 0.05).

The stability of the strongest inhibitors of both recombinant and native GTD, BL5.2, BL6.2, and their monovalent counterparts, BL5.1 and BL6.1, was further assessed and compared to evaluate their potential utility for oral delivery and gastrointestinal functionality. After exposure to simulated GI conditions (pH 1.2–6.8), both monovalent and homodivalent V_H_Hs retained 80%–90% binding capacity (Figure 2a). However, with the addition of pepsin, binding capacity decreased to 50% for BL5.2 and 20% for BL6.2. Nevertheless, the homodivalent constructs were, significantly more stable compared to their respective monovalent counterparts (*p ≤ 0.05) (Figure 2b).

Thermal stability assessment indicated that the monovalent V_H_Hs experienced a 20% reduction in binding across all tested temperatures. In contrast, the homodivalent V_H_Hs showed a smaller decrease in binding, demonstrating a greater stability (*p ≤ 0.05) compared to their monovalent counterparts (Figure 2c). The melting temperature (*T*_m_) for each of the homodivalent V_H_Hs was determined to 74°C for BL5.2 and 71°C for BL6.2, confirming robust thermostability (Table 2). Finally, preliminary tests on shelf‐life and compatibility with milk at 4°C showed a less than 20% reduction in binding capacity for both V_H_Hs formats (Figure 2d).

**TABLE 2:** Melting temperatures (*T*_m_) for the selected V_H_Hs.

V_H_H	*T*_m_ (°C)
BL5.2	74 ± 0.4
BL6.2	71.4 ± 0.2

*Note*: *T*_m_ values calculated for BL5.2 and BL6.2, obtained using PTSA and Boltzmann‐derived denaturation midpoint equation. Data represent averages from triplicate measurements.

Additionally, we conducted a preliminary assessment comparing the intrinsic stability of the homodivalent V_H_H constructs with two commercially available orally delivered antibody‐based products, which utilize IgY and IgG as active components, under identical stress conditions (temperature, pH, and pepsin). The homodivalent V_H_H constructs showed equivalent stability, maintaining their structure as evidenced by intact epitope detection similar to the commercial products (see Figure S2).

### 2.6 Divalent V_H_H constructs display broad cross‐reactivity against nine TcdB toxinotypes

**FIGURE 3:**
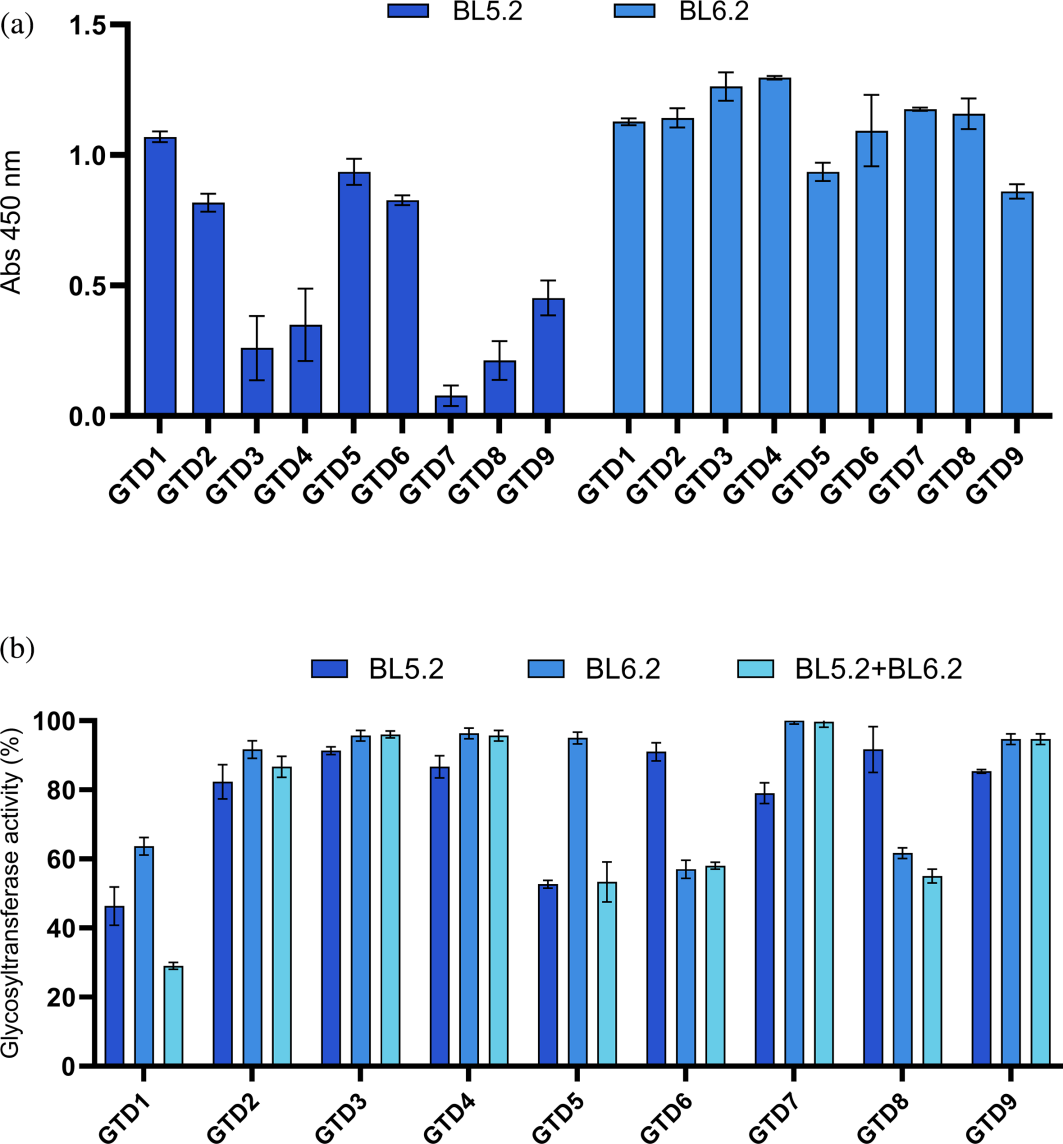
Assessment of cross‐reactivity of selected homodivalent V_H_Hs across GTDs variants from TcdB toxinotypes. (a) Assessment of binding capacity towards nine GTD variants (GTD1–9). Results are based on measurements from two independent experiments, both in duplicates with SD for each group plotted as error bars. (b) In vitro evaluation of the inhibitory effect of the homodivalent V_H_Hs against the enzymatic activity of recombinant GTD variants (GTD1–9) using a glycosyltransferase activity assay. The graph shows the percentage of GTD activity in the presence of each individual homodivalent V_H_H, alone or in combination, relative to the enzymatic activity of GTD in the absence of V_H_H. The molar ratios used were 1:10 (GTD:‘V_H_H binding sites’, i.e., approximately 1:5 GTD:‘homodivalent V_H_H’) and approximately 1:5 (GTD:‘V_H_H binding sites’) when combined. The graph (b) represents the mean of three technical replicates with SD for each group plotted as error bars.

We evaluated the binding and inhibitory effects of the homodivalent V_H_Hs BL5.2 and BL6.2 against GTD variants from nine TcdB toxinotypes (Table S1) using ELISA and a glycosyltransferase activity assay. BL6.2 exhibited broader binding (Figure 3a) and was able to inhibit the variants GTD1, GTD6, and GTD8 by 35%–40%. Meanwhile, BL5.2 showed less cross‐reactive binding, but inhibited the activity of the variants GTD1 and GTD5 by approximately 50%, as well as GTD7 by 20% (Figure 3). Recognizing the individual binding and inhibition properties of the homodivalent V_H_Hs, we explored their combined effect and observed synergistic inhibition of GTD1, reducing the activity by 70%, when mixed (Figure 3b).

### 2.7 BL5.1 and BL6.1 bind to distinct epitopes near the catalytic site

**FIGURE 4:**
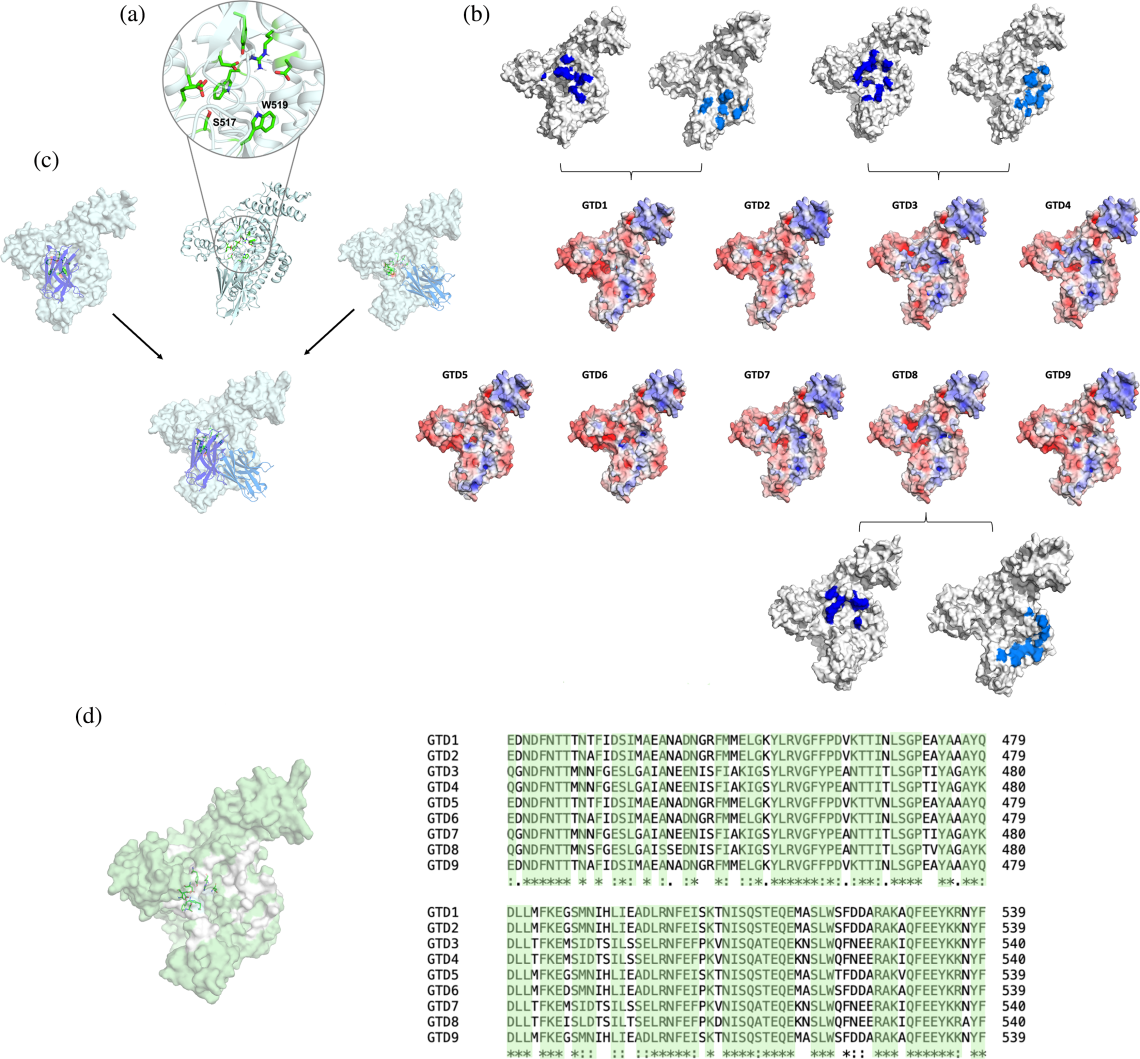
Epitope characterization of nine GTD variants binding to the monovalent BL5.1 and BL6.1. (a) Close‐up of the catalytic site of GTD, highlighting the residues involved in UDP‐glu coordination in green. The catalytic site is overlapping with the binding sites of BL5.1 and BL6.1. (b) Electrostatic potential (ranging from −5 to 5; red indicates negative, and blue indicates positive) calculated with Adaptative Poisson–Boltzmann Solver (APBS) mapped on the surface of the nine GTD variants. The respective interaction sites of BL5.1 (marine) and BL6.1 (blue) with GTD1, GTD3, and GTD8 are projected on the surface. (c) Representative binding poses of BL5.1 and BL6.1 to GTD1, depicting the different binding areas of both V_H_Hs close to the catalytic site. (d) Sequence conservation of the nine GTD variants. The conserved residues are highlighted in pale green in both the structural representation as well as in the sequence alignment. GTD sequences were based on the toxinotypes reported by Mansfield et al. (2020).

To study the binding interactions between BL5.1 and BL6.1 and the nine GTD variants, we predicted the potential interaction sites using ColabFold and AMBER 22. We obtained five distinct binding poses of each V_H_H to the GTD variants. Based on these starting models, we performed molecular dynamics (MD) simulations to characterize the respective interaction sites. First, we explored the catalytic site of the GTD domain, focusing on the residues involved in interaction with its substrate (uridine diphosphate glucose; UDP‐glu) (Figure 4a). The calculated electrostatic potentials demonstrate that all nine GTDs differ in their surface properties close to the catalytic site, probably co‐determining the changes in V_H_H binding and glycosyltransferase activity (Figure 4b). Exploring the interaction sites between BL5.1 and BL6.1 and the GTD variants revealed distinct and non‐overlapping interaction sites close to the catalytic site, potentially explaining the strong additive effect when combined in an in vitro setting (Figure 3b, GTD1). This hypothesis is strengthened by the representative binding poses of BL5.1 and BL6.1, which appear to allow co‐binding to GTD (Figure 4c). Further, sequence conservation analysis revealed that while most of the GTD sequences are highly conserved, especially the area around the catalytic site reveals the highest variations, affecting mainly the epitope targeted by BL5.1. These observations, together with the cross‐reactivity evaluation, prompted the development of a heterodivalent V_H_H construct BL5–6.2, composed by BL5.1 and BL6.1 connected by a (GGGGS)_3_ linker.

### 2.8 Primary and secondary bile salts influence binding of the heterodivalent BL5–6.2 to TcdB

**FIGURE 5:**
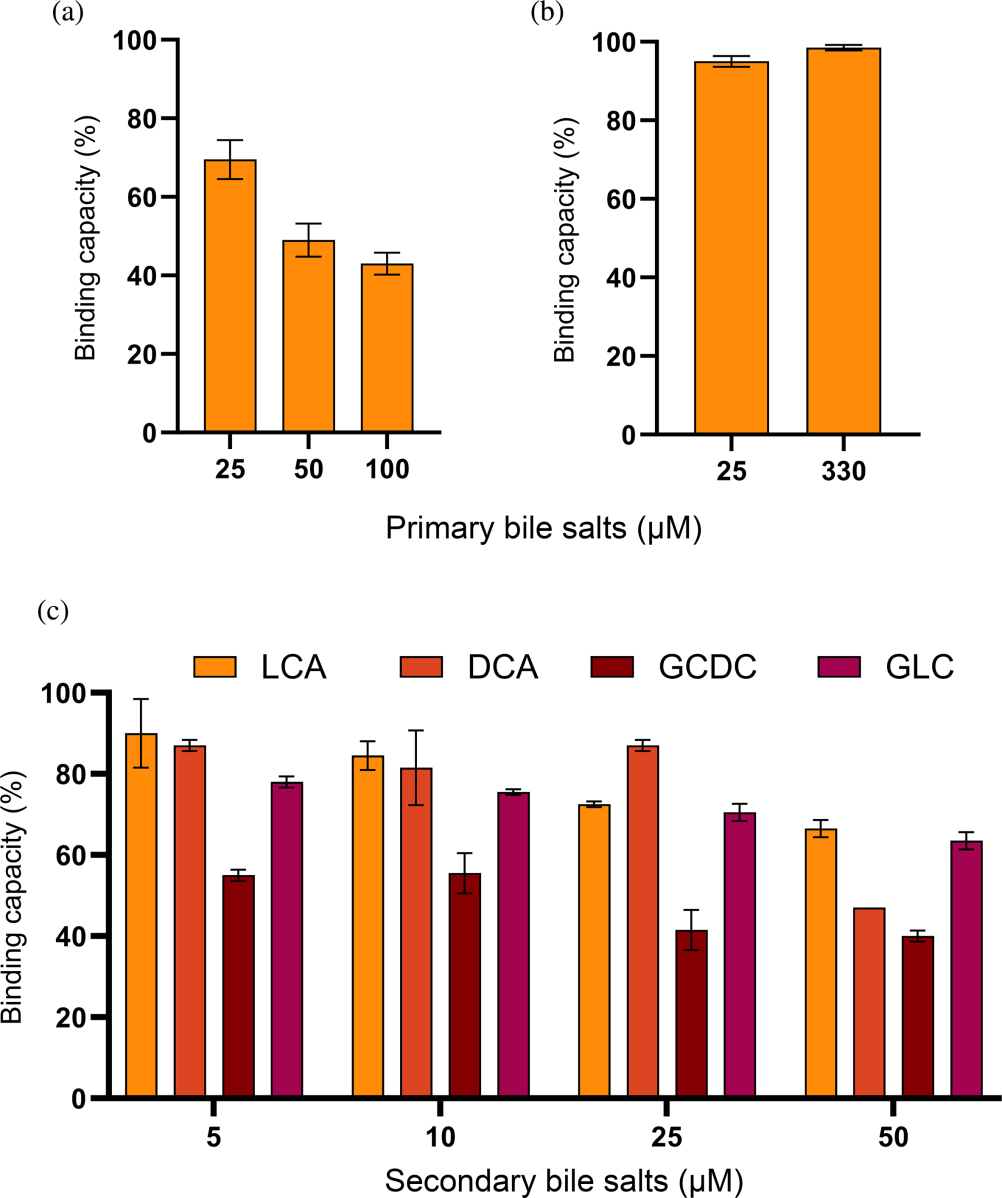
Assessment of the effect of bile salts on the ability of heterodivalent BL5–6.2 to bind to native TcdB. (a) Evaluation of the influence of primary bile salts on the binding capacity of BL5–6.2 towards native TcdB. The graph shows the binding of BL5–6.2 to TcdB in the presence of primary bile salts (25–100 μM), relative to a positive control (TcdB alone). (b) Assessment of the influence of primary bile salts on the binding capacity of BL5–6.2 towards recombinant GTD binding. The graph shows the binding of BL5–6.2 to GTD in the presence of primary bile salts (25–330 μM), relative to a positive control (GTD alone). (c) Assessment of the influence of secondary bile salts; glycodeoxycholate (GCDC), deoxycholic acid (DCA), glycolithocholate (GLC), and lithocholic acid (LCA) on the binding capacity of BL5–6.2 towards native TcdB. The graph shows the binding of BL5–6.2 to native TcdB in the presence of the four secondary bile salts (5–50 μM), relative to a positive control (TcdB alone). The graphs (a‐c) represent the mean of two independent experiments, each in duplicates with SD for each group plotted as error bars.

We evaluated the binding properties of heterodivalent BL5–6.2 to TcdB and GTD in the presence of primary and secondary bile salts. Primary bile salts decreased TcdB binding by 30%–55% (Figure 5a) in a concentration‐dependent manner, with no significant impact to recombinant GTD binding at very high concentration (330 μM) (Figure 5b). With respect to the four secondary bile salts, 5 μM glycodeoxycholate (GCDC) reduced binding between BL5–6.2 and TcdB by 45% (Figure 5c). At the highest concentration (50 μM) of individual secondary bile salts, deoxycholic acid (DCA) reduced the binding signal by 50%, whereas glycolithocholate (GLC) and lithocholic acid (LCA) only caused a 35% and 30% decrease, respectively (Figure 5c).

### 2.9 The heterodivalent V_H_H construct inhibits TcdB‐mediated cytotoxicity in human colonic cells

We conducted in vitro assays using HCA‐7 (human colonic adenocarcinoma) cell monolayers to evaluate BL5–6.2's ability to inhibit TcdB‐induced cytotoxicity.

**FIGURE 6:**
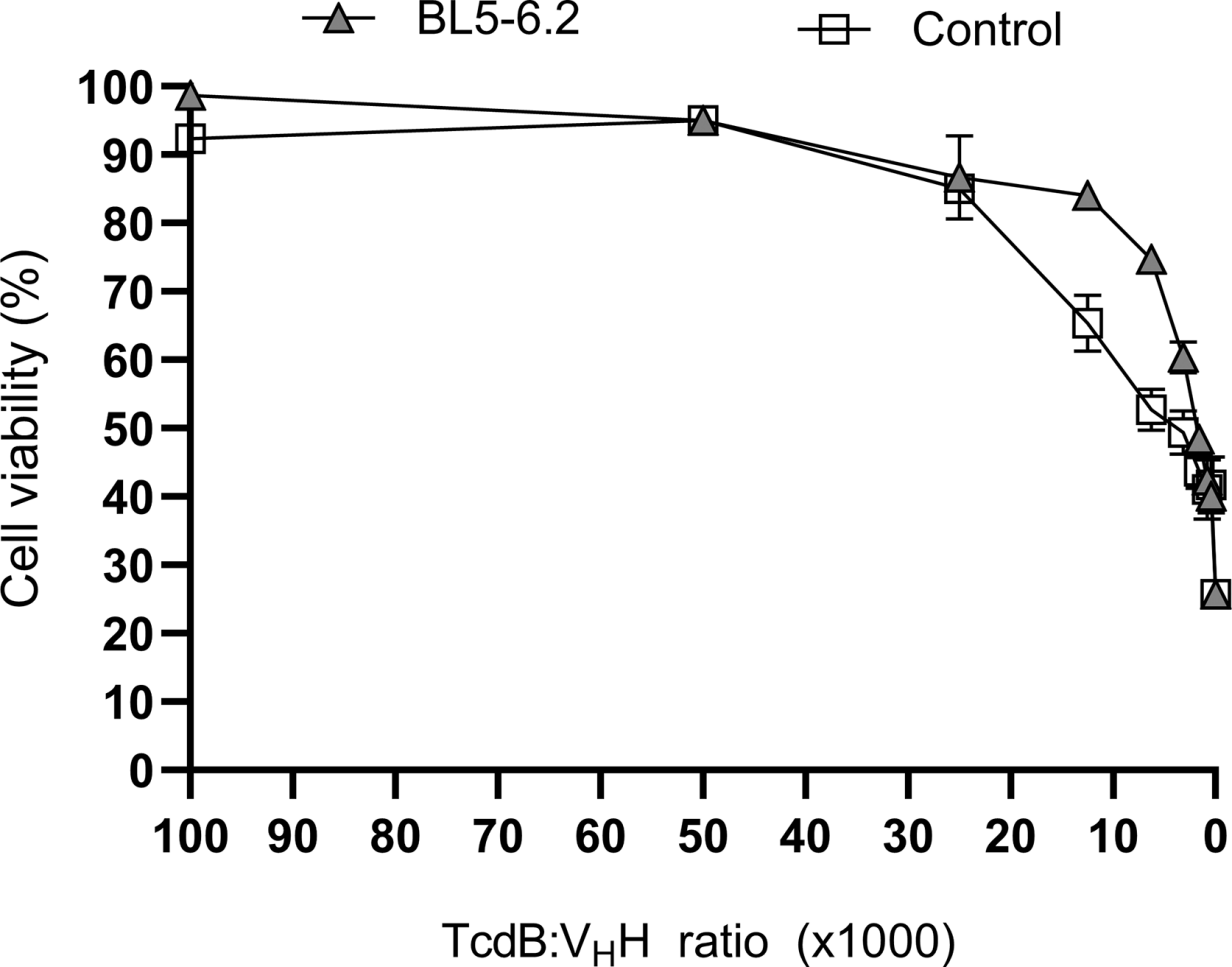
Cell cytotoxic assay evaluation of the neutralization capacity of BL5–6.2 against TcdB. Assessment of the cell viability of HCA‐7 cells after incubation with TcdB and varying concentrations of BL5–6.2 (triangles) or V_H_H control (squares), compared to a group of non‐treated cells using a luminescent cell viability assay. The graph shows cell viability (%) as calculated by measuring the ATP levels. The graphs represent the mean of triplicates with SD for each group plotted as error bars. Results are from three independent experiments.

For comparison, TcdB inhibition assays were conducted using a known TcdB‐neutralizing control V_H_H (Yang et al., 2014) along with the BL5–6.2 construct. TcdB alone induced cell rounding at 10 ng/mL and significant cytotoxicity beyond 50 ng/mL (data not shown). Interestingly, even a fivefold increase in TcdB did not achieve LD_100_, a response also observed in other cell lines (D'Auria et al., 2015). To challenge the heterodivalent V_H_H construct we tested its inhibitory capacity using 100 ng/mL of TcdB. At a molecular ratio of 1:30,000 (TcdB:‘V_H_H binding sites’), both BL5–6.2 and V_H_H control protected approximately 90% of the cells (Figure 6). However, BL5–6.2 was able to protect 60% of the cells down to a molar ratio of 1:3000 (TcdB:‘V_H_H binding sites’). The experimental IC_50_ values were comparable, with BL5–6.2 exhibiting a marginally lower IC_50_ (3.06 nM) than the control V_H_H (3.6 nM) (Table 3).

**TABLE 3:** IC_50_ values for two V_H_Hs against TcdB measured in an in vitro cell viability assay.

V_H_H	IC_50_ (nM)	*R* ^2^
BL5–6.2	3.06 ± 0.11	0.98
Benchmark	3.6 ± 0.15	0.96

*Note*: Half‐maximal inhibitory concentration (IC_50_) was calculated using non‐linear regression and dose–response inhibition models using GraphPad Prism.

## 3 DISCUSSION

Here, we present a systematic in vitro methodology tailored to evaluate the developability properties of homo‐ and heterodivalent V_H_H constructs for GI applications. Our approach focuses on assessing the stability of these constructs and their effectiveness in neutralizing TcdB from *C. difficile*, a toxin acting in the complex environment of the colon. An important aspect of our approach is the use of early screening for neutralization of both the recombinant and native GTD from TcdB to ensure that the identification of optimal V_H_Hs was not only based on target affinity, but also on functionality. After rigorous developability assessment, the homodimers BL5.2 and BL6.2 were demonstrated to be promising candidates for oral delivery. Yet, further development of the heterodivalent V_H_H construct, BL5–6.2, led to the most promising TcdB‐neutralizing candidate. This was confirmed by its inhibitory potency against TcdB, which is comparable to a well‐characterized control V_H_H in an HCA‐7 cell cytotoxicity assay (Yang et al., 2014).

Artificial intelligence‐based tools, such as AlphaFold2 (AF2) and RoseTTAFold, have revolutionized protein structure prediction, as evidenced by recent studies (Baek et al., 2021; Tunyasuvunakool et al., 2021). The combination of structural in silico tools, such as ColabFold (Mirdita et al., 2022) and AMBER 22 (Case et al., 2023), allowed for the prediction of non‐overlapping epitopes and interpretation of the synergistic inhibition observed by BL5.2 and BL6.2. The epitope targeted by BL6.2 is more conserved among the GTD variants compared to that of BL5.2, which could explain why the heterodivalent V_H_H construct displays both high affinity to and broader coverage of toxinotypes. This in silico analysis provides molecular insights into why the heterodivalent V_H_H construct, BL5‐6.2, was identified as the best TcdB‐neutralizing candidate.

The utility of orally delivered V_H_Hs, and derived constructs, as toxin inhibitors, lies in their intrinsically high stability under various extreme conditions (i.e., high temperature, low pH, proteolytic environment, and high salt concentrations) combined with their high target specificity (Arbabi Ghahroudi et al., 1997; Debatis et al., 2023; Fiil et al., 2022; Goldman et al., 2017; Petersson et al., 2023). Another key feature of V_H_Hs is their extended complementary determining regions (in particular, the CDR3), which enable them to interact with difficult‐to‐reach epitopes within certain antigens.The GTD from TcdB is an example of such an epitope, as it is partially shielded by the CROPS region at physiological pH (7.4) and undergoes two conformational changes during the toxin's mechanisms of action (Chen et al., 2019; Liu et al., 2022; Stijlemans et al., 2004). Taking the target antigen's mechanism of action into consideration can be of great benefit when assessing the developability of a particular V_H_H, as illustrated in this study, where we show the effect of GTD cleavage from the TcdB holotoxin mediated by conformational changes. Mimicking the GTD‐release process in vitro, we observed that our homodivalent V_H_H displayed a reduced ability to inhibit the native GTD compared to the monovalent V_H_H against the recombinant GTD under static conditions. This highlights the need to incorporate such parameters during the discovery process. In contrast, the only clinically approved human monoclonal antibody for prevention of recurrent CDI, bezlotoxumab (Zinplava), targets the CROPS region of TcdB and was specifically developed for intravenous administration. Consequently, stability parameters relevant for GI applications have not been critical developability criteria for bezlotoxumab, and entirely different epitopes may be available for binding monoclonal antibodies or V_H_H constructs during oral delivery and intravenous administration, respectively (Lee et al., 2017; Navalkele & Chopra, 2018).

To date, multiple studies on the development of antibody‐based strategies intended to block *C. difficile* toxins in situ have been performed. Some studies have focused on oral delivery of full‐size polyclonal antibody mixtures derived from immunized animals (Hutton et al., 2017; Kink & Williams, 1998; Lyerly et al., 1991; Roberts et al., 2020; Van Dissel et al., 2005). This strategy has shown to be effective in neutralizing TcdB in vivo, but due to a lack of target specificity and fluctuating titers of neutralizing antibodies in the polyclonal mixture, standardizing the approach has been challenging (Roberts et al., 2020). The inherent batch‐to‐batch variation for polyclonal antibodies derived from the plasma of immunized animals can, however, be overcome by employing precision fermentation and recombinant DNA technology (Ferrara et al., 2015; Nielsen et al., 2010). Moreover, the use of recombinant DNA technology also allows for the expression of engineered antibody formats that can be optimized to possess high developability (Fernández‐Quintero et al., 2023), such as the V_H_H. In this relation, other studies have explored the development and utility of various V_H_H constructs, ranging from simple formats to larger multimeric constructs, for blocking TcdB and TcdA in the GI tract (Andersen et al., 2016; Hussack, Hirama, et al., 2011; Schmidt et al., 2016).

A relevant aspect when assessing the developability of V_H_Hs for oral delivery and GI applications involves assessing their functionality. This includes their ability to bind and inhibit their target after ingestion and exposure to the GI environment, but also comparing their intrinsic stability with that of other commercial antibody‐based products, such as IgY and IgG. It is crucial to recognize that the two benchmark products examined in this study comprise non‐specific antibody mixtures purified from either egg yolk (IgY) or bovine colostrum (IgG). Consequently, their stability assessment was focused on structural integrity, unlike the V_H_Hs reported here, which are evaluated for binding capacity retention. Moreover, both products are formulated as finished goods, which enhances their stability, whereas our V_H_Hs were assessed as pure protein, indicating that V_H_H stability can be improved in a final formulation. The GI environment is characterized by extreme pH variations and high proteolytic activity, which impact toxin conformation and availability, which in turn may affect the target binding ability and neutralization capacity of a V_H_H selected under different conditions (Hussack, Arbabi‐Ghahroudi, et al., 2011; Maffey et al., 2016; Rutten et al., 2012; Shinozaki et al., 2017). We consistently show that the discovered V_H_Hs are stable under GI conditions to the same extent as, or exceeding, the investigated commercial antibody products. However, another factor that is seldomly considered is the presence of bile. Bile salts, integral for dietary fat digestion and absorption in the small intestine (Chiang & Ferrell, 2018), have diverse roles, including the regulation of cellular homeostasis and the modulation of gut bacterial growth (Larabi et al., 2023). Moreover, bile salts can alter protein structure and function, and are known to induce a compact conformation of TcdB (Aguirre et al., 2021; Cremers et al., 2014; Nair, 1976; Tam et al., 2020; Winston & Theriot, 2016; Zhuang et al., 2017). However, the impact of bile on the binding and inhibitory capacity of V_H_Hs towards TcdB remains relatively unexplored. While alternative approaches, such as subjecting V_H_Hs to gastric juice or chyme, can provide broader insights into V_H_H stability under more complex GI conditions (Debatis et al., 2023; Fiil et al., 2022; Harmsen et al., 2006), the direct influence of bile salts might be underestimated when assessing how fit‐for‐purpose TcdB‐targeting V_H_Hs are. In our experiments, we observed a concentration‐dependent effect on V_H_H binding capacity for both primary and secondary bile salts. This suggests that bile‐induced structural changes in TcdB may reduce epitope accessibility, a phenomenon not observed when working with recombinant GTD alone. Secondary bile salts, predominantly found in the distal part of the large intestine, play a more significant role in the natural environment, in which TcdB exerts its effects (Zeng et al., 2019). It is important to note that unconjugated DCA, constituting 90% of the secondary bile salts in the distal colon (Chiang & Ferrell, 2018; Hamilton et al., 2007), caused the least reduction in the binding capacity of the heterodivalent V_H_H construct. Additionally, despite challenges in measuring the exact concentrations of secondary bile salts due to their variability and the dynamic nature of the GI environment, we believe that the highest concentrations we tested (50 and 100 μM) exceed those reported for natural conditions, thus, likely representing a worst‐case scenario for the V_H_Hs tested (Beuers & Pusl, 2004; Di Ciaula et al., 2017). Various physiological, dietary, hormonal, and pathological factors, including gut microbiota, influence primary bile salt production and metabolism in the GI tract, with implications for secondary bile salt conversion (Di Ciaula et al., 2017; Guzior & Quinn, 2021; Zeng et al., 2019). Therefore, investigating the concentration dependence of bile salt concentration on binding between antigen‐V_H_H can be a critical parameter for developability profiling.

Combining two or more V_H_Hs in one polypeptide chain can result in a protein construct with enhanced functionality due to increased avidity and potentially broader epitope coverage (Schmidt et al., 2016; Yang et al., 2014). This is particularly relevant for *C. difficile* where several clinically relevant TcdB toxinotypes have been reported, with distinct differences in protein sequence (Mansfield et al., 2020). Other studies have reported synergistic inhibitory effects by the combination of V_H_Hs specific towards distinct TcdB toxinotypes (Zhao et al., 2021). In this context, integrating an evaluation of cross‐reactivity in our developability assessment led to the construction of the heterodivalent BL5–6.2, which binds various toxinotypes, and thus may find broader application than less cross‐reactive constructs.

The work herein presents a systematic approach for assessing the developability and utility of orally delivered V_H_H constructs designed to neutralize TcdB in the GI environment. However, replicating the complexity of the GI environment in vitro is challenging, and limitations remain. Future work could focus on incorporating more complex GI models, emulating digestion and intestinal transit. Yet with currently available models, the ability to recover samples for analysis of V_H_H‐toxin binding and inhibition remains limited (Minekus, 2015; Thuenemann et al., 2015). Additionally, while the discovered heterodivalent V_H_H construct, BL5–6.2, shows promising properties in vitro for inhibiting TcdB in simulated GI environments, clinical research is needed to fully assess the robustness of the in vitro approach employed here, and how useful the heterodivalent V_H_H construct is in vivo.

## 4 CONCLUSION

Taken together, these findings demonstrate how homo‐ and heterodivalent V_H_H constructs can be evaluated early in the discovery process for their suitability for oral delivery and GI applications, that is, their developability in relation to oral administration and enteric effect. This approach emphasizes the inclusion of relevant, yet often overlooked, parameters affecting binding and functional neutralization of the V_H_H constructs (e.g., the effect of bile salts). Furthermore, our results highlight the importance of utilizing a tailored in vitro developability assessment focusing on both target binding, but importantly also toxin inhibition; this is particularly true for V_H_H constructs targeting antigens with a complex biochemistry and mechanism of action, such as TcdB, which undergoes critical conformational changes during its course of action in vivo. By performing such a holistic characterization and assessment with an end in mind focus, V_H_H constructs that are fit‐for‐purpose can more efficiently be discovered and developed.

## 5 MATERIALS AND METHODS

### 5.1 Immunogens and immunization

The gene encoding GTD (amino acid residues M1‐542) of TcdB from strain VPI10463 was cloned into the pSANG10 vector with a C‐terminal 6xHis‐tag and subsequently transformed into *Escherichia coli* strain BL21 (DE3) (New England BioLabs, USA) for expression. The overexpression protocol was adapted from Ahmadi et al. (2020). The His‐tagged GTD was purified using Ni‐NTA agarose (ThermoFisher, R90110, USA) in a 1× PBS buffer with 20 mM imidazole, followed by elution using a high‐imidazole buffer (1× PBS + 250 mM imidazole). Size exclusion chromatography was performed on the eluted fractions using a HiLoad 16/600 Superdex 75 pg column (Cytiva, USA). Protein purity and concentration were determined using SDS‐PAGE gel electrophoresis and absorbance at 280 nm, respectively, with corrections made for molecular weight and extinction coefficient (60,530) calculated using the Expasy Protparameter server.

Two alpacas were immunized with the purified recombinant GTD at the VIB Nanobody core facility in Brussels. Immunizations adhered to the National Health Law's guidelines for laboratory animal care and use. The alpacas received up to five subcutaneous injections at weekly intervals, each consisting of 160 μg of GTD and Gerbu adjuvant P. At 4 and 8 days post the final injection, approximately 100 mL of anticoagulated blood was drawn from each animal for the extraction of peripheral blood lymphocytes (PBLs), which served as a source for V_H_H genetic material.

### 5.2 Construction of V_H_H libraries and panning

Two independent V_H_H libraries were generated from the immunized alpacas. Total RNA was isolated from the collected PBLs at both 4 and 8 days post‐final injection. The RNA from each time point was combined for each alpaca, which then served as a template for cDNA synthesis. About 50 μg of this combined RNA was used to synthesize the first strand of cDNA using an oligo (dT) primer. V_H_H encoding sequences were amplified from this cDNA, digested using *Pst*I and *Not*I restriction enzymes, and then cloned into the similarly digested phagemid vector pMECS (Vincke et al., 2012). Employing the protocol by Griffiths et al. (1993), three panning rounds were conducted on antigen‐coated solid phases (100 μg/mL in 100 mM NaHCO_3_, pH 8.2) (Griffiths et al., 1993). The first panning round was done individually for each library. Afterwards, outputs from both libraries were pooled, amplified, and the phages were purified for the succeeding panning rounds. After each round, the enrichment of antigen‐specific phages was ascertained by contrasting the number of phagemid particles eluted from antigen‐coated wells with those eluted from negative controls.

### 5.3 Antigen biotinylation

The antigens, recombinant GTD or TcdB, were biotinylated using a molar ratio of 1:2 (antigen:biotin) as described by Laustsen et al. (2018). The modified biotin used for the process was EZ‐Link NHS‐PEG4‐Biotin (ThermoFisher, A39259, USA).

### 5.4 Normalized binding screening

The outputs corresponding to the second and third phage panning round were independently subcloned into the expression vector pSANG10‐3F which has a C‐terminal 6xHis‐3xFlag‐tag. This was achieved through restriction enzyme digestion (*Pst*I and *Not*I) and ligation. Resulting constructs were then transformed into *E. coli* strain BL21 (DE3) (New England BioLabs, USA). A total of 376 individual colonies were expressed in 96‐well plates and the supernatant was used for normalized binding screening using dissociation‐enhanced lanthanide fluorescence immunoassay (DELFIA) as described by Laustsen et al. (2018). In brief, individual colonies were inoculated into 150 μL of LB + Km (50 μg/mL) medium per well in a 96‐well polypropylene microtiter plate (Greiner, Bio‐One, Germany). The plate was incubated at 30°C with constant agitation at 800 rpm and 80% humidity overnight (O/N). Five microliters of the O/N culture was transferred to a 96‐well plate containing 150 μL of autoinduction media (Formedium, AIMTB0210, UK) and incubated for 20 h at 30°C, 800 rpm with 80% humidity. Additionally, black 96‐well Mainor plates (ThermoScientific, 7605, USA) were coated O/N at 4°C with an anti‐Flag antibody (Sigma‐Aldrich, F3165, USA) at a concentration of 2.5 μg/mL (60 μL per well). The plates were washed three times with 1× PBS containing 0.1% Tween followed by three times with 1× PBS and then blocked O/N using 3% milk in 1× PBS (MPBS). The supernatant from the cultures was recovered by centrifugation. After washing the plates, 30 μL of the supernatant, mixed with 30 μL of 6% MPBS, was added to the wells and incubated for 1 h at RT. Following an additional washing step, 100 μg of biotinylated GTD diluted in 3% MPBS, was added to the plate and incubated for 1 h at RT. Bound antigen was detected using 0.2 ng/μL of europium‐conjugated streptavidin diluted in DELFIA assay buffer (Perkin Elmer, USA), followed by an enhancement solution (Perkin Elmer, USA). The signal readout was performed by TRF with 320 nm excitation and 615 nm emission wavelengths.

Based on the signal intensity, the top‐performing clones were selected and sequenced using the SecqF primer (5′GGAGATATACATATGAAATACCTGC). The DNA and amino acid sequences of the V_H_H were aligned to compare the CDR's groups using CLC Main Workbench (QIAGEN, Netherlands).

### 5.5 Protein expression

The genes of interest were cloned into the expression vector pSANG10 for GTD or pSANG10‐3F for monovalent, homodivalent, or heterodivalent V_H_H constructs, and expressed in the *E. coli* strain BL21 (DE3) (New England BioLabs, USA) following the protocol reported by Ahmadi et al. (2020). Briefly, a single colony of the relevant construct was inoculated in LB + Km (50 μg/mL) medium and incubated at 37°C O/N. The following day, 1 L autoinduction medium was inoculated using the O/N cultures and incubated O/N at 30°C, 200 rpm. The cells were harvested by centrifugation at 4300 × *g* for 10 min, and the pellet re‐suspended in 50 mL TES buffer (30 mM Tris–HCl pH 8.0, 1 mM EDTA, 20% sucrose (w/v)) containing 25 U/mL of Benzonase Nuclease (ThermoFisher, 88700, USA) Protease Inhibitor Cocktail (Roche, 11836145001, Switzerland) and 1.5 mg of r‐lysozyme (Sigma‐Aldrich, 12650‐88‐3, USA) per gram of cell pellet. After 20 min of incubation on ice, the cells were centrifuged at 4300 × *g* for 10 min and supernatant was recovered. The cell pellet was re‐suspended in 50 mL of 5 mM MgSO_4_ supplemented with the same reagents as before and incubated on ice for 20 min. After centrifugation at 4300 × *g* for 10 min, supernatant was pooled with the supernatant from the previous step. The pooled supernatants were centrifuged once again at 30,000 × *g* for 30 min for removal of remaining cell debris and non‐lysed cells. Purification of the His‐tagged proteins from the supernatant was performed using Ni‐NTA agarose (ThermoFisher, R90110, USA). The eluted fractions underwent size exclusion chromatography using a HiLoad 16/600 Superdex 75 pg column (Cyntiva, USA). Protein purity was assessed through SDS‐page gel electrophoresis. Protein concentration was determined at 280 nm and corrected for molecular weight and extinction coefficient (32,100 and 60,360 for monovalent and homodivalent V_H_H, respectively) calculated using the Expasy ProtParam server.

### 5.6 Screening for inhibitory effect of V_H_H using glycosyltransferase activity assay

To evaluate the inhibitory effect of the V_H_H on the glycosyltransferase activity of recombinant GTD, we modified the method detailed by Brown et al. (2012). Individual V_H_Hs (or negative control without V_H_H) were mixed with 100 μg of GTD in 1× PBS in a molar ratio of 1:20 (GTD:V_H_H monovalent) and incubated for 1 h at 37°C. After incubation, the samples were transferred to a 96‐well plate, clear, flat bottom, non‐binding surface (Cayman Chemical, 400014, USA) and mixed with assay buffer (50 mM HEPES pH 7.4, 150 mM KCl, 5 mM MgCl_2_, 0.5 nM NADH). Components including: 0.5 mM phosphoenolpyruvate (PEP), 1 and 1.5 units of pyruvate kinase/lactate dehydrogenase (PK/LDH) and 2 nM UDP‐glucose were added, achieving a final volume of 200 μL per well. In this assay, the activity of glucosyltransferase (GTD) on UDP‐glucose leads to a series of reactions, culminating in the oxidation of NADH to NAD, which was tracked by measuring absorbance at 340 nm. The reaction was monitored by measuring the absorbance every 30 s for up to 3 h (until NADH was fully consumed).

Inhibition of glycosyltransferase activity was calculated using the linear part of the absorbance curves, setting the slope of the GTD (control) curve to 100%, and calculating the V_H_H‐inhibited activity as the slope rendered by an individual V_H_H construct relative to the control.

### 5.7 Enzyme‐linked immunosorbent assay (ELISA)

96‐well Maxisorp plates (Nunc, Italy) were coated overnight at 4°C with 2.5 μg/mL of either antigen GTD or TcdB (strain VPI10463, TNAC, CBD‐TNL, UK) in 1× PBS. The following day, the plates were washed (three times with 1× PBS containing 0.1% Tween followed by three times with 1× PBS). After washing, the plates were blocked with 3% M‐PBS for 1 h at RT after which washing was repeated. Immediately, treated, or untreated monovalent, homodivalent V_H_H (500 nM) were added. As control, the human IgG bezlotoxumab was used. Plates incubated for 1 h at RT after which the plates were washed again. Immediately, the secondary anti‐Flag M2‐peroxidase (HRP) antibody (Sigma‐Aldrich, A8592, USA) or anti‐human IgG‐Fc‐HRP (ThermoFisher, A10648) was added in a ratio of 1:20,000 vol:vol in 3% M‐PBS. The plates were incubated for 1 h at RT followed by washing, and the peroxidase reaction was initiated by adding the substrate, 3,3′,5,5′‐tetramethylbenzidine (TMB) in peroxide solution. The plates were incubated until color development (typically 5–15 min), at which point the reaction was stopped with 2 M H_3_PO_4_. The absorbance at 450 nm was measured and the ratio between absorbances (treated:untreated) was presented as percent binding relative to untreated samples.

### 5.8 Measurement of kinetic parameters using BLI and SPR

The kinetic parameters were determined in collaboration with National Biologics Facility (NBF, Denmark). The kinetic parameters of: BL6.1, BL7.1, BL6.2, and BL7.2 were determined using BLI on the Octet system (Sartorius, Germany). A concentration of 50 mM biotinylated recombinant GTD toxin was used for V_H_H capture to a streptavidin (SA) biosensor (Sartorius, 10‐0009, Germany). The ligand‐loaded biosensors were then immersed in serial dilutions of each individual monovalent or homodivalent V_H_H in 1× kinetic buffer (Sartorius, 181105, Germany) ranging from 0.2 to 200 nM. The association time and dissociation time were set to 180 and 3600 s respectively. Data curves acquired were analyzed using the Octet Analysis Studio software from Sartorius to calculate the kinetic *k*_on_, *k*_off_, and local and global *K*_D_. The kinetic parameters of BL5.1 and BL5.2 were determined using SPR on a Biacore 8K system (Cytiva, USA) through the single‐cycle kinetic method. GTD at 10 μg/mL was immobilized using the Biotin CAPture kit (Cyntiva, 28920234, USA). Serial dilutions (0.1–100 nM) of BL5.1 and BL5.2 were injected sequentially with an association of 400 s and a final dissociation time of 7,000 s at a flow rate of 30 μL/min. Data curves were analyzed using Biacore Insight evaluation software (Cyntiva) to calculate the kinetic *k*_on_, *k*_off_, and global *K*_D_. The mean residence time (*T*_R_) was calculated in minutes based on the formula *T*_R_ = 1/*k*_off_, as described by Rodríguez‐Rodríguez et al. (2016).

### 5.9 Reformatting to homo‐ and heterodivalent V_H_H constructs

The genes corresponding to V_H_H‐(Gly4Ser)3‐V_H_H were cloned into the vector pSANG10‐3F, with a C‐terminal 6xHis‐3xFlag‐tag. The resulting vectors were transformed into the *E. coli* strain BL21 (DE3) (New England BioLabs, USA) for protein expression. The expression and purification processes were performed as mentioned in Section 5.5.

### 5.10 Blocking native TcdB‐GTD

To verify release of GTD from the holotoxin through auto‐processing (GTD is only active when cleaved), TcdB (strain VPI10463, TNAC, CBD‐TNL, UK) was treated as described by Chung et al. (2018). Briefly, 2 μg of TcdB was incubated with 100 μM inositol hexakisphosphate (InsP_6_) (Sigma‐Aldrich, 68388, USA) in a 20 μL reaction buffer (20 mM Tris, 150 mM NaCl, pH 7.4) for 1 h at 37°C. The reaction was stopped by addition 5 μL of 5× Laemmli buffer and immediate transfer to a heating block at 95°C incubating for 10 min. Autocleavage analysis was performed using SDS‐PAGE. To investigate blocking, native TcdB (50 μg) was mixed with individual homodivalent V_H_H constructs at a 1:20 ratio (TcdB:binding sites in homodivalent V_H_H) and incubated for 15 min at 37°C. After incubation, auto‐cleavage was induced by addition of 200 μM of InsP_6_, followed by a 30‐min incubation. Removal of InsP_6_ and buffer exchange was done by transferring 100 μL of each sample to individual 10 kDa Amicon ultra 0.5 centrifuge tubes (Sigma‐Aldrich, UFC5010, USA) and performing two rounds of dia‐filtration, each time adding 400 μL of activity buffer without NADH (50 mM Hepes pH 7.4, 150 mM KCl, 5 mM MgCl_2_) before reducing the volume to the initial 100 μL. After reaching 100 μL final volume, the remaining components of the reaction were added: 0.5 mM phosphoenolpyruvate (PEP), 1 and 1.5 units of pyruvate kinase/lactate dehydrogenase (PK/LDH), 2 nM UDP‐glucose and 0.5 mM NADH, achieving a final volume of 200 μL per well. Following, the samples were analyzed in the glycosyltransferase assay as previously described in Section 5.6.

### 5.11 Stability at GI‐relevant pH

The stability at relevant pH conditions was assessed for each monovalent and homodivalent V_H_H construct, by preparing 1.5 μM V_H_H construct in the following solutions: 1× PBS pH 7.4 (untreated samples), simulated gastric fluid (SGF; 35 mM NaCl pH 1.2), simulated intestinal fluid (SIF; 50 mM KH_2_PO_4_ pH 6.8), and 50 mM MES pH 5.5, prepared according to recommended USP standards and as reported by Maffey et al. (2016). All samples were incubated for a minimum of 60 min at 37°C. Additionally, certain samples were treated with 1 mg/mL equivalent to 100 U/mL of pepsin (Sigma‐Aldrich, P7000) in SGF and incubated for 60 min at 37°C. Post‐incubation, the SGF sample's pH was neutralized by adding 70 μL of 200 mM Na_2_CO_3_. Each V_H_H's residual binding capacity was assessed via ELISA, with results compared to untreated samples and then converted to binding percentage.

### 5.12 Thermostability, *T*_m_ measurements, and shelf‐life stability

Each monovalent and homodivalent V_H_H construct was subjected to varied temperatures: 500 nM concentrations were exposed to temperatures between 37 and 80°C for 1 h. Following these treatments, binding efficiency against GTD was evaluated using ELISA, with data compared to untreated samples and transformed to binding percentage. Protein Thermal Shift™ assays were conducted using the Protein Thermal Shift Dye kit (Applied Biosystems, 4461146, USA) and the QuantStudio™ 6 Pro real‐time PCR instrument (Applied Biosystem, USA). The melting point (*T*_m_) determination was executed by the Protein Thermal Shift Software (Applied Biosystems, USA). The assay's final volume was 20 μL, comprising 1× PBS, Protein Thermal Shift Dye (3×), and 10 μg of purified proteins. A control without protein was also run. Temperature was ramped from 25 to 99°C, incrementing at 0.05°C/s, with each scan performed in triplicate. The V_H_H's unfolding temperatures (Boltzmann *T*_m_) were deduced from the melting curves' inflection points using the Protein Thermal Shift Software.

Shelf stability was assessed by diluting 500 nM of monovalent or homodivalent V_H_H in 3% skimmed milk and submitted to HTST pasteurization process (90°C for 1 s, then returned to room temperature for 3 min, followed by cooling at 4°C for 5 min), after that the samples were stored at 4°C for 7 days. Binding capacity was evaluated by ELISA against GTD.

### 5.13 Stability comparison of V_H_H constructs with commercial orally delivered antibody‐based products

The intrinsic stability of the homodivalent V_H_H constructs was assessed and compared to that of IgY‐based (Hyperimmune Egg Powder, i26) and IgG‐based orally delivered products (Mega IgG2000, Microbiome Labs). This was evaluated by incubating 0.250 mg/mL of each protein independently in the following solutions: 1× PBS at pH 7.4 (untreated samples), SGF (35 mM NaCl, pH 1.2) with and without 1 mg/mL pepsin, equivalent to 100 U/mL (Sigma‐Aldrich, P7000), SIF (50 mM KH_2_PO_4_, pH 6.8), and 50 mM MES pH 5.5 for 1 h with shaking (120 rpm). These solutions were prepared in accordance with USP standards and as reported by Maffey et al. (2016). Additionally, the proteins were exposed to temperatures ranging from 37 to 80°C for 1 h with 120 rpm agitation. Following all treatments, the integrity of each protein was evaluated using a direct ELISA. The treated samples were directly coated (26.6 μg/mL) onto 96‐well MaxiSorp plates (Nunc, Italy) for 1 h at 4°C. Plates were washed five times with 1× PBS containing 0.1% Tween‐20. After washing, the plates were blocked with 3% M‐PBS for 1 h at room temperature (RT); washing was then repeated. Subsequently, secondary antibodies were added: Protein A‐HRP (GenScript, M00089, USA), rabbit anti‐chicken IgY‐HRP (Invitrogen, 31401, USA), and anti‐bovine IgG‐HRP (Sigma‐Aldrich, A5295, USA) for the detection of the V_H_H constructs, IgY, and IgG, respectively, at a dilution of 1:20,000 (vol/vol) in 1% M‐PBS. The plates were incubated for 1 h at RT, followed by washing. The peroxidase reaction was initiated by adding the substrate 3,3′,5,5′‐tetramethylbenzidine (TMB) in a peroxide solution. The plates were incubated until color development was observed, at which point, the reaction was stopped with 2 M H_3_PO_4_. The absorbance at 450 nm was then measured.

### 5.14 Assessment of cross‐reactivity against GTD variants using ELISA

The genes encoding the GTD (residues M1‐542) from 9 of the 12 toxinotypes of TcdB relevant clades (Table S1) were cloned into the vector pSANG10 for expression and purification as described in Section 5.5. Both binding and blocking capabilities against these recombinant GTD variants were evaluated using ELISA and glycosyltransferase activity assays (detailed in Section 5.6), but with a molecular ratio of 1:10 (GTD: binding sites in homodivalent V_H_H). The resultant values were analyzed and converted into percentages to facilitate comparisons as mentioned in previous sections.

### 5.15 Prediction of BL5.1 and BL6.1 binding epitopes

ColabFold, that integrates AF2 and RoseTTAFold with the rapid homology search capability of MMseqs2, was used for prediction of the homo‐ and heteromeric complexes with a quality on par with AF2 and AF‐multimer (Mirdita et al., 2022). Five binding poses were predicted for BL5.1 and BL6.1 to the nine GTD variants. Starting from these five binding poses, we performed three repetitions of 100 ns of classical MD simulations for each pose using the AMBER 22 simulation software package which contains the pmemd.cuda module (Case et al., 2023). The structure models were placed into cubic water boxes of TIP3P water molecules with a minimum wall distance to the protein of 12 Å as recommended by El Hage et al. (2018) and Jorgensen et al. (1983). Parameters for all simulations were derived from the AMBER force field 14SB according to Bayly et al. (1995) and Maier et al. (2015). To neutralize the charges, we used uniform background charges based on those reported by Darden et al. (1993), Hub et al. (2014) and Salomon‐Ferrer et al. (2013). Each system was carefully equilibrated using a multistep equilibration protocol (Wallnoefer et al., 2010). Bonds involving hydrogen atoms were restrained using the SHAKE algorithm, allowing a timestep of 2.0 fs (Andersen, 1983). The systems' pressure was maintained at 1 bar by applying weak coupling to an external bath using the Berendsen algorithm reported by Berendsen et al. (1984). The Langevin Thermostat was utilized to keep the temperature at 300 K during the simulations as described in Adelman and Doll (1976).

For the MD analysis, we first calculated the electrostatic surface potentials using APBS for all nine GTD variants to identify differences in biophysical properties. Then, we used the MD simulations for all nine GTD variants in complex with BL5.1 and BL6.1 and calculated the respective contacts using the GetContacts software (Stanford University, https://getcontacts.github.io). We computed the interactions within one protein structure, and the different protein interfaces. Simultaneously we monitored the evolution of contacts during the simulation. In parallel we quantified the contacts of the different poses and performed a cluster analysis of the hierarchical agglomerative clustering implemented in cpptraj to identify the most probable representative binding poses as reported by Jurrus et al. (2018) and Roe and Cheatham (2013).

### 5.16 Bile salt stability assays

Biotinylated native TcdB and recombinant GTD were prepared at concentrations of 25 nM. They were incubated at 37°C for 30 min with varying concentrations of a mixture of primary salts (USP standards, 1071304, USA), and individual secondary bile salts: LCA, DCA, GLC, and GCDC (all sourced from Sigma‐Aldrich, USA). Post‐incubation, the binding capacity of the V_H_H construct was assessed using capture DELFIA (as described in Section 5.4). Data from these evaluations were analyzed and converted into percentages for comparison purposes.

### 5.17 Cell viability assay

Human colonic adenocarcinoma cell lines (HCA‐7) were cultured under standard conditions using Dulbecco's Modified Eagle's Medium supplemented with 10% fetal bovine serum (FBS) (HyClone, SH30071.03, USA) and 1% pen‐strep (DMEM) (Biowest, France). These were cultivated in 75 cm^2^ cell culture flasks at 37°C in an environment with 5% CO_2_ until they achieved roughly 80% confluency. Cells were recovered by trypsinization and transferred to 96‐well culture plates at a density of 0.01 × 10^6^ cells/well, whereas 24‐well plates were seeded with 0.05 × 10^6^ cells/well, followed by a 24‐h incubation. The susceptibility of HCA‐7 cells to TcdB was assessed using a dose–response assay. Concentrations of TcdB spanning from 0.010 to 500 ng/mL were investigated. After rinsing cells with 1× PBS, varying TcdB concentrations (diluted in growth medium) were added to each well, followed by a 48‐h incubation. Cells in 24‐well plates underwent a visual assessment, whereas cell viability in the 96‐well plates was determined using the CellTiter‐Glo cell viability kit (Promega, G7570, USA). Percentage of cell viability was calculated by comparing the treated cell with untreated.

Blocking activity was evaluated by pre‐mixing 100 ng/mL of TcdB with the heterodivalent V_H_H construct and a V_H_H control (Yang et al., 2014) in 1:2 serial dilutions at ratios spanning from 100,000 to 391:1 (binding sites V_H_H:TcdB) prior to application on the cells. Cell viability was measured using the mentioned kit, with results compared to cells that remained untreated. Data were then converted into percentages. As negative control, cells were incubated in the presence of the highest concentrations of V_H_H constructs. No significative effect on cell viability was observed compared with untreated cells.

### 5.18 Half‐maximal inhibitory concentration

To determine the half‐maximal inhibitory concentration (IC_50_), we conducted a dose–response cell viability assay, varying the concentration of the V_H_H from 3.5 μM to 0 through serial dilutions. The obtained values were then utilized to calculate the IC_50_ using a non‐linear regression model based on the Hill‐slope equation.

### 5.19 Data processing and visualization

GraphPad Prism version 10.2.2 was used for figure generation and all statistical analyses. Data were analyzed using the unpaired Mann–Whitney test, in which differences were considered significant (*) at *p* values of ≤0.05 and highly significant (**) at *p* values of ≤0.01. Average values and standard deviations were calculated after transforming the values to the figure scale illustrated. CLC Main Workbench version 23.0.2 was used for sequence analysis and alignment. DNA and protein sequences were analyzed using the Omega clustering algorithm using CLC workbench software (Qiagen, Germany). Structural visualization was performed using Pymol Molecular Graphics System, Version 2.5.2, Schrödinger, LLC.

## AUTHOR CONTRIBUTIONS

**Everardo R. Rodriguez Rodriguez:** Methodology; investigation; visualization; writing – review and editing; writing – original draft. **Rune Thorbjørn Nordvang:** Methodology; supervision; writing – review and editing; project administration. **Marcus Petersson:** Writing – original draft; writing – review and editing. **Jakob Kræmmer Haar Rendsvig:** Writing – review and editing; investigation. **Emma Wenzel Arendrup:** Investigation; visualization; writing – review and editing. **Monica L. Fernández Quintero:** Investigation; visualization; writing – review and editing. **Timothy P. Jenkins:** Investigation; visualization; writing – review and editing. **Andreas H. Laustsen:** Conceptualization; methodology; writing – original draft; resources; funding acquisition; writing – review and editing. **Sandra Wingaard Thrane:** Conceptualization; methodology; writing – review and editing; funding acquisition; resources; project administration; supervision; writing – original draft.

## CONFLICT OF INTEREST STATEMENT

S.W.T and A.H.L. are co‐founders, employees, and shareholders in Bactolife A/S. E.R.R.R., R.T.N., M.P., and J.K.H.R. are employees of Bactolife A/S.

## Supporting information


**Appendix S1:** Supporting information.

## References

[pro5035-bib-0001] Adelman SA , Doll JD . Generalized Langevin equation approach for atom/solid‐surface scattering: general formulation for classical scattering off harmonic solids. J Chem Phys. 1976;64(6):2375–2388.

[pro5035-bib-0002] Aguirre AM , Yalcinkaya N , Wu Q , Swennes A , Tessier ME , Roberts P , et al. Bile acid‐independent protection against *Clostridioides difficile* infection. PLoS Pathog. 2021;17(10):e1010015. 34665847 10.1371/journal.ppat.1010015 PMC8555850

[pro5035-bib-0003] Ahmadi S , Pucca MB , Jürgensen JA , Janke R , Ledsgaard L , Schoof EM , et al. An in vitro methodology for discovering broadly‐neutralizing monoclonal antibodies. Sci Rep. 2020;10(1):1–7. 32612183 10.1038/s41598-020-67654-7 PMC7329857

[pro5035-bib-0004] Aktories K , Schwan C , Jank T . *Clostridium difficile* toxin biology. Annu Rev Microbiol. 2017;71:281–307. 28657883 10.1146/annurev-micro-090816-093458

[pro5035-bib-0005] Andersen HC . Rattle: a “velocity” version of the shake algorithm for molecular dynamics calculations. J Comput Phys. 1983;52(1):24–34.

[pro5035-bib-0006] Andersen KK , Strokappe NM , Hultberg A , Truusalu K , Smidt I , Mikelsaar RH , et al. Neutralization of *Clostridium difficile* toxin B mediated by engineered lactobacilli that produce single‐domain antibodies. Infect Immun. 2016;84(2):395–406. 26573738 10.1128/IAI.00870-15 PMC4730582

[pro5035-bib-0007] Arbabi Ghahroudi M , Desmyter A , Wyns L , Hamers R , Muyldermans S . Selection and identification of single domain antibody fragments from camel heavy‐chain antibodies. FEBS Lett. 1997;414(3):521–526. 9323027 10.1016/s0014-5793(97)01062-4

[pro5035-bib-0008] Ausserwöger H , Schneider MM , Herling TW , Arosio P , Invernizzi G , Knowles TPJ , et al. Non‐specificity as the sticky problem in therapeutic antibody development. Nat Rev Chem. 2022;6(12):844–861. 37117703 10.1038/s41570-022-00438-x

[pro5035-bib-0009] Baek M , DiMaio F , Anishchenko I , Dauparas J , Ovchinnikov S , Lee GR , et al. Accurate prediction of protein structures and interactions using a three‐track neural network. Science. 2021;373(6557):871–876. 34282049 10.1126/science.abj8754 PMC7612213

[pro5035-bib-0010] Bailly M , Mieczkowski C , Juan V , Metwally E , Tomazela D , Baker J , et al. Predicting antibody developability profiles through early stage discovery screening. mAbs. 2020;12(1):1743053. 32249670 10.1080/19420862.2020.1743053 PMC7153844

[pro5035-bib-0011] Bayly CI , Merz KM , Ferguson DM , Cornell WD , Fox T , Caldwell JW , et al. A second generation force field for the simulation of proteins, nucleic acids, and organic molecules. J Am Chem Soc. 1995;117(19):5179–5197.

[pro5035-bib-0012] Behring E . Untersuchungen über das Zustandekommen der Diphtherie‐Immunität und der Tetanus‐Immunität bei Thieren. Deutsch Med Wochenshr. 1890;16(49):1113–1114.

[pro5035-bib-0013] Berendsen HJC , Postma JPM , Van Gunsteren WF , Dinola A , Haak JR . Molecular dynamics with coupling to an external bath. J Chem Phys. 1984;81(8):3684–3690.

[pro5035-bib-0014] Beuers U , Pusl T . Bile salts and their metabolism. Encyclopedia of Biological Chemistry. Amsterdam: Elsevier; 2004. p. 159–163.

[pro5035-bib-0015] Brown C , Leijon F , Bulone V . Radiometric and spectrophotometric in vitro assays of glycosyltransferases involved in plant cell wall carbohydrate biosynthesis. Nat Prot. 2012;7(9):1634–1650. 10.1038/nprot.2012.089 22899332

[pro5035-bib-0016] Carter GP , Chakravorty A , Nguyen TAP , Mileto S , Schreiber F , Li L , et al. Defining the roles of TcdA and TcdB in localized gastrointestinal disease, systemic organ damage, and the host response during *Clostridium difficile* infections. MBio. 2015;6(3):e00551. 26037121 10.1128/mBio.00551-15 PMC4453007

[pro5035-bib-0017] Case DA , Aktulga HM , Belfon K , Cerutti DS , Cisneros GA , Cruzeiro VWD , et al. AmberTools. J Chem Inf Model. 2023;63(20):6183–6191. 37805934 10.1021/acs.jcim.3c01153 PMC10598796

[pro5035-bib-0018] Chen P , Lam K h , Liu Z , Mindlin FA , Chen B , Gutierrez CB , et al. Structure of the full‐length *Clostridium difficile* toxin B. Nat Struct Mol Biol. 2019;26(8):712–719. 31308519 10.1038/s41594-019-0268-0 PMC6684407

[pro5035-bib-0019] Chiang JYL , Ferrell JM . Bile acid metabolism in liver pathobiology. Gene Exp. 2018;18(2):71–87. 10.3727/105221618X15156018385515 PMC5954621 29325602

[pro5035-bib-0020] Chung SY , Schöttelndreier D , Tatge H , Fühner V , Hust M , Beer LA , et al. The conserved Cys‐2232 in *Clostridioides difficile* toxin B modulates receptor binding. Front Microbiol. 2018;9:410252. 10.3389/fmicb.2018.02314 PMC6212469 30416488

[pro5035-bib-0021] Cremers CM , Knoefler D , Vitvitsky V , Banerjee R , Jakob U . Bile salts act as effective protein‐unfolding agents and instigators of disulfide stress in vivo. PNAS. 2014;111(16):E1610–E1619. 24706920 10.1073/pnas.1401941111 PMC4000791

[pro5035-bib-0022] Darden T , York D , Pedersen L . Particle mesh Ewald: an N·log(N) method for Ewald sums in large systems. J Chem Phys. 1993;98(12):10089–10092.

[pro5035-bib-0023] D'Auria KM , Bloom MJ , Reyes Y , Gray MC , Van Opstal EJ , Papin JA , et al. High temporal resolution of glucosyltransferase dependent and independent effects of *Clostridium difficile* toxins across multiple cell types. BMC Microbiol. 2015;15(1):1–10. 25648517 10.1186/s12866-015-0361-4 PMC4323251

[pro5035-bib-0024] Debatis M , Danz H , Tremblay JM , Gaspie K , Kudej RK , Vigdorovich V , et al. Enteric pharmacokinetics of monomeric and multimeric camelid nanobody single‐domain antibodies. PLoS One. 2023;18(11):e0291937. 38011121 10.1371/journal.pone.0291937 PMC10681176

[pro5035-bib-0025] Di Ciaula A , Garruti G , Baccetto RL , Molina‐Molina E , Bonfrate L , Wang DQH , et al. Bile acid physiology. Ann Hepatol. 2017;16:S4–S14. 29080336 10.5604/01.3001.0010.5493

[pro5035-bib-0026] do Vale A , Cabanes D , Sousa S . Bacterial toxins as pathogen weapons against phagocytes. Front Microbiol. 2016;7:172814. 10.3389/fmicb.2016.00042 PMC4734073 26870008

[pro5035-bib-0027] Drozdowski B , Zhou Y , Kline B , Spidel J , Chan YY , Albone E , et al. Generation and characterization of high affinity human monoclonal antibodies that neutralize staphylococcal enterotoxin B. J Immune Based Therap Vaccines. 2010;8(1):1–9. 21176153 10.1186/1476-8518-8-9 PMC3022601

[pro5035-bib-0028] El Hage K , Hédin F , Gupta PK , Meuwly M , Karplus M . Valid molecular dynamics simulations of human hemoglobin require a surprisingly large box size. eLife. 2018;7:e35560. 29998846 10.7554/eLife.35560 PMC6042964

[pro5035-bib-0029] Faulstich H , Kirchner K , Derenzini M . Strongly enhanced toxicity of the mushroom toxin α‐amanitin by an amatoxin‐specific Fab or monoclonal antibody. Toxicon. 1988;26(5):491–499. 3188055 10.1016/0041-0101(88)90188-2

[pro5035-bib-0030] Fernández‐Quintero ML , Ljungars A , Waibl F , Greiff V , Andersen JT , Gjølberg TT , et al. Assessing developability early in the discovery process for novel biologics. mAbs. 2023;15(1):2171248. 36823021 10.1080/19420862.2023.2171248 PMC9980699

[pro5035-bib-0031] Ferrara F , D'Angelo S , Gaiotto T , Naranjo L , Tian H , Gräslund S , et al. Recombinant renewable polyclonal antibodies. mAbs. 2015;7(1):32. 25530082 10.4161/19420862.2015.989047 PMC4622072

[pro5035-bib-0032] Fiil BK , Thrane SW , Pichler M , Kittilä T , Ledsgaard L , Ahmadi S , et al. Orally active bivalent V_H_H construct prevents proliferation of F4^+^ enterotoxigenic *Escherichia coli* in weaned piglets. iScience. 2022;25(4):104003. 35310945 10.1016/j.isci.2022.104003 PMC8931358

[pro5035-bib-0033] Garcia‐Rodriguez C , Razai A , Geren IN , Lou J , Conrad F , Wen WH , et al. A three monoclonal antibody combination potently neutralizes multiple botulinum neurotoxin serotype E subtypes. Toxins. 2018;10(3):105. 29494481 10.3390/toxins10030105 PMC5869393

[pro5035-bib-0034] Gavrilaș S , Ursachi CȘ , Perța‐Crișan S , Munteanu FD . Recent trends in biosensors for environmental quality monitoring. Sensors. 2022;22(4):1513. 35214408 10.3390/s22041513 PMC8879434

[pro5035-bib-0035] Ghazaei C . Advances in the study of bacterial toxins, their roles and mechanisms in pathogenesis. Malays J Med Sci. 2022;29(1):4–17. 35283688 10.21315/mjms2022.29.1.2 PMC8887987

[pro5035-bib-0036] Goldman ER , Liu JL , Zabetakis D , Anderson GP . Enhancing stability of camelid and shark single domain antibodies: an overview. Front Immunol. 2017;8:1. 28791022 10.3389/fimmu.2017.00865 PMC5524736

[pro5035-bib-0037] Griffiths AD , Malmqvist M , Marks JD , Bye JM , Embleton MJ , McCafferty J , et al. Human anti‐self antibodies with high specificity from phage display libraries. EMBO J. 1993;12(2):725–734. 7679990 10.1002/j.1460-2075.1993.tb05706.x PMC413258

[pro5035-bib-0038] Guzior DV , Quinn RA . Review: microbial transformations of human bile acids. Microbiome. 2021;9(1):1–13. 34127070 10.1186/s40168-021-01101-1 PMC8204491

[pro5035-bib-0039] Hamilton JP , Xie G , Raufman JP , Hogan S , Griffin TL , Packard CA , et al. Human cecal bile acids: concentration and spectrum. Am J Physiol Gastrointest Liver Physiol. 2007;293(1):256–263. 10.1152/ajpgi.00027.2007 17412828

[pro5035-bib-0040] Harmsen MM , Van Solt CB , Van Zijderveld‐Van Bemmel AM , Niewold TA , Van Zijderveld FG . Selection and optimization of proteolytically stable llama single‐domain antibody fragments for oral immunotherapy. Appl Microbiol Biotechnol. 2006;72(3):544–551. 16450109 10.1007/s00253-005-0300-7

[pro5035-bib-0041] Harris B . Exploiting antibody‐based technologies to manage environmental pollution. Trends Biotechnol. 1999;17(7):290–296. 10370236 10.1016/s0167-7799(99)01308-6

[pro5035-bib-0042] He X , Sun X , Wang J , Wang X , Zhang Q , Tzipori S , et al. Antibody‐enhanced, fc gamma receptor‐mediated endocytosis of *Clostridium difficile* toxin a. Infect Immun. 2009;77(6):2294–2303. 19307220 10.1128/IAI.01577-08 PMC2687358

[pro5035-bib-0043] Hortigüela MJ , Wall JG . Improved detection of domoic acid using covalently immobilised antibody fragments. Marine Drugs. 2013;11(3):881–895. 23493076 10.3390/md11030881 PMC3705377

[pro5035-bib-0044] Hub JS , De Groot BL , Grubmüller H , Groenhof G . Quantifying artifacts in Ewald simulations of inhomogeneous systems with a net charge. J Chem Theory Comput. 2014;10(1):381–390. 26579917 10.1021/ct400626b

[pro5035-bib-0045] Hussack G , Arbabi‐Ghahroudi M , Van Faassen H , Songer JG , Ng KKS , MacKenzie R , et al. Neutralization of *Clostridium difficile* toxin A with single‐domain antibodies targeting the cell receptor binding domain. J Biol Chem. 2011;286(11):8961–8976. 21216961 10.1074/jbc.M110.198754 PMC3058971

[pro5035-bib-0046] Hussack G , Hirama T , Ding W , MacKenzie R , Tanha J . Engineered single‐domain antibodies with high protease resistance and thermal stability. PLoS One. 2011;6(11):e28218. 22140551 10.1371/journal.pone.0028218 PMC3227653

[pro5035-bib-0047] Hutton ML , Cunningham BA , Mackin KE , Lyon SA , James ML , Rood JI , et al. Bovine antibodies targeting primary and recurrent *Clostridium difficile* disease are a potent antibiotic alternative. Sci Rep. 2017;7(1):1–13. 28623367 10.1038/s41598-017-03982-5 PMC5473923

[pro5035-bib-0048] Jorgensen WL , Chandrasekhar J , Madura JD , Impey RW , Klein ML . Comparison of simple potential functions for simulating liquid water. J Chem Phys. 1983;79(2):926–935.

[pro5035-bib-0049] Jurrus E , Engel D , Star K , Monson K , Brandi J , Felberg LE , et al. Improvements to the APBS biomolecular solvation software suite. Protein Sci. 2018;27(1):112–128. 28836357 10.1002/pro.3280 PMC5734301

[pro5035-bib-0050] Kang TH , Seong BL . Solubility, stability, and avidity of recombinant antibody fragments expressed in microorganisms. Front Microbiol. 2020;11:552011. 10.3389/fmicb.2020.01927 PMC7546209 33101218

[pro5035-bib-0051] Kink JA , Williams JA . Antibodies to recombinant *Clostridium difficile* toxins A and B are an effective treatment and prevent relapse of *C. difficile*‐associated disease in a hamster model of infection. Infect Immun. 1998;66(5):2018–2025. 9573084 10.1128/iai.66.5.2018-2025.1998 PMC108158

[pro5035-bib-0052] Larabi AB , Masson HLP , Bäumler AJ . Bile acids as modulators of gut microbiota composition and function. Gut Microb. 2023;15(1):2172671. 10.1080/19490976.2023.2172671 PMC9904317 36740850

[pro5035-bib-0053] Laustsen AH , Karatt‐Vellatt A , Masters EW , Arias AS , Pus U , Knudsen C , et al. In vivo neutralization of dendrotoxin‐mediated neurotoxicity of black mamba venom by oligoclonal human IgG antibodies. Nat Commun. 2018;9(1):1–9. 30279409 10.1038/s41467-018-06086-4 PMC6168529

[pro5035-bib-0054] Lee Y , Lim WI , Bloom CI , Moore S , Chung E , Marzella N . Bezlotoxumab (Zinplava) for *Clostridium difficile* infection: the first monoclonal antibody approved to prevent the recurrence of a bacterial infection. Pharm Ther. 2017;42(12):735–738. PMC5720485 29234211

[pro5035-bib-0055] Liu J , Kothe M , Zhang JJ , Oloo E , Stegalkina S , Mundle ST , et al. Novel structural insights for a pair of monoclonal antibodies recognizing non‐overlapping epitopes of the glucosyltransferase domain of *Clostridium difficile* toxin B. Curr Res Struct Biol. 2022;4:96–105. 35469152 10.1016/j.crstbi.2022.03.003 PMC9034018

[pro5035-bib-0056] Lyerly DM , Bostwick EF , Binion SB , Wilkins TD . Passive immunization of hamsters against disease caused by *Clostridium difficile* by use of bovine immunoglobulin G concentrate. Infect Immun. 1991;59(6):2215–2218. 2037383 10.1128/iai.59.6.2215-2218.1991 PMC257992

[pro5035-bib-0057] Maffey L , Vega CG , Miño S , Garaicoechea L , Parreño V . Anti‐VP6 VHH: an experimental treatment for rotavirus A‐associated disease. PLoS One. 2016;11(9):e0162351. 27603013 10.1371/journal.pone.0162351 PMC5014449

[pro5035-bib-0058] Maier JA , Martinez C , Kasavajhala K , Wickstrom L , Hauser KE , Simmerling C . ff14SB: improving the accuracy of protein side chain and backbone parameters from ff99SB. J Chem Theory Comput. 2015;11(8):3696–3713. 26574453 10.1021/acs.jctc.5b00255 PMC4821407

[pro5035-bib-0059] Mansfield MJ , Tremblay BJM , Zeng J , Wei X , Hodgins H , Worley J , et al. Phylogenomics of 8,839 *Clostridioides difficile* genomes reveals recombination‐driven evolution and diversification of toxin A and B. PLoS Pathog. 2020;16(12):e1009181. 33370413 10.1371/journal.ppat.1009181 PMC7853461

[pro5035-bib-0060] Minekus M . The TNO gastro‐intestinal model (TIM). The impact of food bioactives on health: in vitro and ex vivo models. Cham: Springer; 2015. p. 37–46. 29787039

[pro5035-bib-0061] Mirdita M , Schütze K , Moriwaki Y , Heo L , Ovchinnikov S , Steinegger M . ColabFold: making protein folding accessible to all. Nat Methods. 2022;19(6):679–682. 35637307 10.1038/s41592-022-01488-1 PMC9184281

[pro5035-bib-0062] Morens DM . Antibody‐dependent enhancement of infection and the pathogenesis of viral disease. Clin Infect Dis. 1994;19(3):500–512. 7811870 10.1093/clinids/19.3.500

[pro5035-bib-0063] Nair PP . Bile–salt–protein interactions. The bile acids: chemistry, physiology, and metabolism. Boston: Springer; 1976. p. 29–52.

[pro5035-bib-0064] Navalkele BD , Chopra T . Bezlotoxumab: an emerging monoclonal antibody therapy for prevention of recurrent *Clostridium difficile* infection. Biol Targets Ther. 2018;12:11. 10.2147/BTT.S127099 PMC5779312 29403263

[pro5035-bib-0065] Nielsen LS , Baer A , Müller C , Gregersen K , Mønster NT , Rasmussen SK , et al. Single‐batch production of recombinant human polyclonal antibodies. Mol Biotechnol. 2010;45(3):257–266. 20306237 10.1007/s12033-010-9270-9 PMC2881207

[pro5035-bib-0066] Orrell KE , Zhang Z , Sugiman‐Marangos SN , Melnyk RA . *Clostridium difficile* toxins A and B: receptors, pores, and translocation into cells. Crit Rev Biochem Mol Biol. 2017;52(4):461–473. 28545305 10.1080/10409238.2017.1325831

[pro5035-bib-0067] Petersson M , Thrane SW , Gram L , Muyldermans S , Laustsen AH . Orally delivered single‐domain antibodies against gastrointestinal pathogens. Trends Biotechnol. 2023;41(7):875–886. 36774206 10.1016/j.tibtech.2023.01.015

[pro5035-bib-0068] Pitiot A , Heuzé‐Vourc'h N , Sécher T . Alternative routes of administration for therapeutic antibodies—state of the art. Antibodies. 2022;11(3):56. 36134952 10.3390/antib11030056 PMC9495858

[pro5035-bib-0069] Pruitt RN , Chambers MG , Ng KKS , Ohi MD , Lacy DB . Structural organization of the functional domains of *Clostridium difficile* toxins A and B. PNAS. 2010;107(30):13467–13472. 20624955 10.1073/pnas.1002199107 PMC2922184

[pro5035-bib-0070] Reilly RM , Domingol R , Sandhu J . Oral delivery of antibodies future pharmacokinetic trends. Clin Pharmacokinet. 1997;32(4):313–323. 9113439 10.2165/00003088-199732040-00004

[pro5035-bib-0071] Roberts AK , Harris HC , Smith M , Giles J , Polak O , Buckley AM , et al. A novel, orally delivered antibody therapy and its potential to prevent *Clostridioides difficile* infection in pre‐clinical models. Front Microbiol. 2020;11:578903. 33072047 10.3389/fmicb.2020.578903 PMC7537341

[pro5035-bib-0072] Rodríguez‐Rodríguez ER , Olamendi‐Portugal T , Serrano‐Posada H , Arredondo‐López JN , Gómez‐Ramírez I , Fernández‐Taboada G , et al. Broadening the neutralizing capacity of a family of antibody fragments against different toxins from Mexican scorpions. Toxicon. 2016;119:52–63. 27212628 10.1016/j.toxicon.2016.05.011

[pro5035-bib-0073] Roe DR , Cheatham TE . PTRAJ and CPPTRAJ: software for processing and analysis of molecular dynamics trajectory data. J Chem Theory Comput. 2013;9(7):3084–3095. 26583988 10.1021/ct400341p

[pro5035-bib-0074] Rouha H , Weber S , Janesch P , Maierhofer B , Gross K , Dolezilkova I , et al. Disarming *Staphylococcus aureus* from destroying human cells by simultaneously neutralizing six cytotoxins with two human monoclonal antibodies. Virulence. 2018;9(1):231–247. 29099326 10.1080/21505594.2017.1391447 PMC5955178

[pro5035-bib-0075] Rutten L , De Haard H , Verrips T . Improvement of proteolytic stability through in silico engineering. Methods Mol Biol. 2012;911:373–381. 22886263 10.1007/978-1-61779-968-6_22

[pro5035-bib-0076] Salomon‐Ferrer R , Case DA , Walker RC . An overview of the Amber biomolecular simulation package. WIREs Comput Mol Sci. 2013;3(2):198–210.

[pro5035-bib-0077] Sánchez‐Zuno GA , Matuz‐Flores MG , González‐Estevez G , Nicoletti F , Turrubiates‐Hernández FJ , Mangano K , et al. A review: antibody‐dependent enhancement in COVID‐19: the not sofriendly side of antibodies. Int J Immunopathol Pharmacol. 2021;35:1–15. 10.1177/20587384211050199 PMC8512237 34632844

[pro5035-bib-0078] Schmidt DJ , Beamer G , Tremblay JM , Steele JA , Kim HB , Wang Y , et al. A tetraspecific VHH‐based neutralizing antibody modifies disease outcome in three animal models of *Clostridium difficile* infection. Clin Vaccine Immunol. 2016;23(9):774–784. 27413067 10.1128/CVI.00730-15 PMC5014919

[pro5035-bib-0079] Shinozaki N , Hashimoto R , Fukui K , Uchiyama S . Efficient generation of single domain antibodies with high affinities and enhanced thermal stabilities. Sci Rep. 2017;7(1):1–11. 28725057 10.1038/s41598-017-06277-x PMC5517631

[pro5035-bib-0080] Sørensen CV , Ledsgaard L , Wildenauer HHK , Dahl CH , Ebersole TW , Bohn MF , et al. Cross‐reactivity trends when selecting scFv antibodies against snake toxins using a phage display‐based cross‐panning strategy. Sci Rep. 2023;13(1):1–10. 37349546 10.1038/s41598-023-37056-6 PMC10287648

[pro5035-bib-0081] Stijlemans B , Conrath K , Cortez‐Retamozo V , Van Xong H , Wyns L , Senter P , et al. Efficient targeting of conserved cryptic epitopes of infectious agents by single domain antibodies. African trypanosomes as paradigm. J Biol Chem. 2004;279(2):1256–1261. 14527957 10.1074/jbc.M307341200

[pro5035-bib-0082] Tam J , Icho S , Utama E , Orrell KE , Gómez‐Biagi RF , Theriot CM , et al. Intestinal bile acids directly modulate the structure and function of *C. difficile* TcdB toxin. Proc Natl Acad Sci U S A. 2020;117(12):6792–6800. 32152097 10.1073/pnas.1916965117 PMC7104382

[pro5035-bib-0083] Thuenemann EC , Giuseppina GM , Rich GT , Faulks RM . Dynamic gastric model (DGM). The impact of food bioactives on health: in vitro and ex vivo models. Cham: Springer; 2015. p. 47–59. 29787039

[pro5035-bib-0084] Torres VVL , Coggon CF , Wells TJ . Antibody‐dependent enhancement of bacterial disease: prevalence, mechanisms, and treatment. Infect Immun. 2021;89(4):e00054‐21. 33558319 10.1128/IAI.00054-21 PMC8090947

[pro5035-bib-0085] Tsubokura K , Berndtson E , Bogstedt A , Kaijser B , Kim M , Ozeki M , et al. Oral administration of antibodies as prophylaxis and therapy in *Campylobacter jejuni*‐infected chickens. Clin Exp Immunol. 2003;108(3):451–455. 10.1046/j.1365-2249.1997.3901288.x PMC1904686 9182891

[pro5035-bib-0086] Tunyasuvunakool K , Adler J , Wu Z , Green T , Zielinski M , Žídek A , et al. Highly accurate protein structure prediction for the human proteome. Nature. 2021;596(7873):590–596. 34293799 10.1038/s41586-021-03828-1 PMC8387240

[pro5035-bib-0087] Van Dissel JT , De Groot M , Hensgens CMH , Numan S , Kuijper EJ , Veldkamp P , et al. Bovine antibody‐enriched whey to aid in the prevention of a relapse of *Clostridium difficile*‐associated diarrhoea: preclinical and preliminary clinical data. J Med Microbiol. 2005;54(2):197–205. 15673517 10.1099/jmm.0.45773-0

[pro5035-bib-0088] Vincke C , Gutiérrez C , Wernery U , Devoogdt N , Hassanzadeh‐Ghassabeh G , Muyldermans S . Generation of single domain antibody fragments derived from camelids and generation of manifold constructs. Methods Mol Biol. 2012;907:145–176. 22907350 10.1007/978-1-61779-974-7_8

[pro5035-bib-0089] Wallnoefer HG , Handschuh S , Liedl KR , Fox T . Stabilizing of a globular protein by a highly complex water network: a molecular dynamics simulation study on factor Xa. J Phys Chem B. 2010;114(21):7405–7412. 20446703 10.1021/jp101654g

[pro5035-bib-0090] Walsh G , Walsh E . Biopharmaceutical benchmarks 2022. Nat Biotechnol. 2022;40(12):1722–1760. 36471135 10.1038/s41587-022-01582-x PMC9735008

[pro5035-bib-0091] Winston JA , Theriot CM . Impact of microbial derived secondary bile acids on colonization resistance against *Clostridium difficile* in the gastrointestinal tract. Anaerobe. 2016;41:44–50. 27163871 10.1016/j.anaerobe.2016.05.003 PMC5050083

[pro5035-bib-0092] Wolf Pérez AM , Lorenzen N , Vendruscolo M , Sormanni P . Assessment of therapeutic antibody developability by combinations of in vitro and in silico methods. Methods Mol Biol. 2022;2313:57–113. 34478132 10.1007/978-1-0716-1450-1_4

[pro5035-bib-0093] Yang Z , Schmidt D , Liu W , Li S , Shi L , Sheng J , et al. A novel multivalent, single‐domain antibody targeting TcdA and TcdB prevents fulminant *Clostridium difficile* infection in mice. J Infect Dis. 2014;210(6):964–972. 24683195 10.1093/infdis/jiu196 PMC4192054

[pro5035-bib-0094] Zeng H , Umar S , Rust B , Lazarova D , Bordonaro M . Secondary bile acids and short chain fatty acids in the colon: a focus on colonic microbiome, cell proliferation, inflammation, and cancer. Int J Mol Sci. 2019;20(5):1214. 30862015 10.3390/ijms20051214 PMC6429521

[pro5035-bib-0095] Zhao H , Tasch M , Dodds M , Gewe M , Martinez A , Hutton M , et al. Using antibody synergy to engineer a high potency biologic cocktail against *C. difficile* . bioRxiv. 2021. 10.1101/2021.12.21.473715

[pro5035-bib-0096] Zhuang S , Li Q , Cai L , Wang C , Lei X . Chemoproteomic profiling of bile acid interacting proteins. ACS Central Sci. 2017;3(5):501–509. 10.1021/acscentsci.7b00134 PMC5445530 28573213

