# Supplementary material for: Fit‐for‐purpose heterodivalent single‐domain antibody for gastrointestinal targeting of toxin B from Clostridium difficile
Source: Protein Sci. 2024 Jun 26;33(7):e5035. doi: 10.1002/pro.5035 (PMC11201815; doi:10.1002/pro.5035)
Supplement: Supplementary file 1 — Appendix S1: Supporting information. [file PRO-33-e5035-s001.docx]

**Supplementary material:**





**Supplementary Figure 1. *In vitro* autoproteolysis of GTD from native TcdB.** This sodium dodecyl sulfate-polyacrylamide gel electrophoresis (SDS-PAGE) shows recombinant GTD (rGTD), native TcdB, and TcdB after incubation with InsP6, which induces GTD autocleavage. The arrow indicates the native GTD.


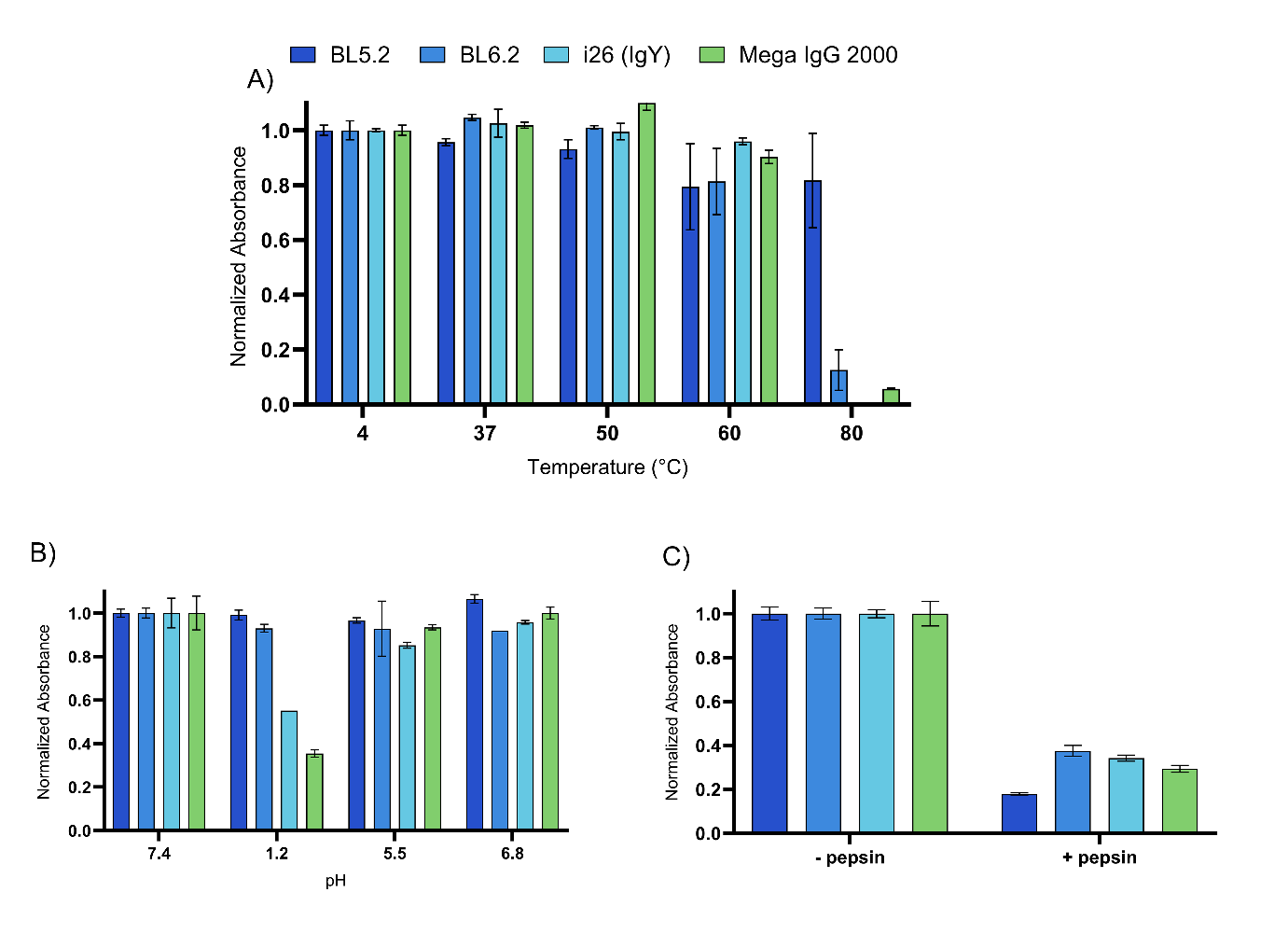


**Supplementary Figure 2. Assessment of intrinsic stability of homodivalent V_H_H constructs and commercial antibody-based products. A)** Assessment of thermostability. The graph illustrates normalized absorbance provided by the detection antibody against the homodivalent V_H_H, IgY, or IgG post 1 hour incubation at temperatures ranging from 37 to 80 °C. Absorbance values that do not decrease can be interpreted as indicative of the proteins maintain their intact conformation. **B)** Assessment of pH stability within a physiologically relevant range (pH 1.2 to 6.8), according to (Maffey et al. 2016). The graph displays normalized absorbance given by the detection antibody against the homodivalent V_H_H, IgY, or IgG after pH exposure compared with the control. **C)** Proteases stability. The graph shows the normalized absorbance given by the detection antibody against each protein after exposure to pepsin (1 mg/mL). The graphs represent normalized means of technical duplicates with with SD for each group plotted as error bars. i26 (IgY) refers to a product containing purified IgY derived from chicken eggs. Mega IgG 2000 denotes concentrated immunoglobulins derived from bovine colostrum.


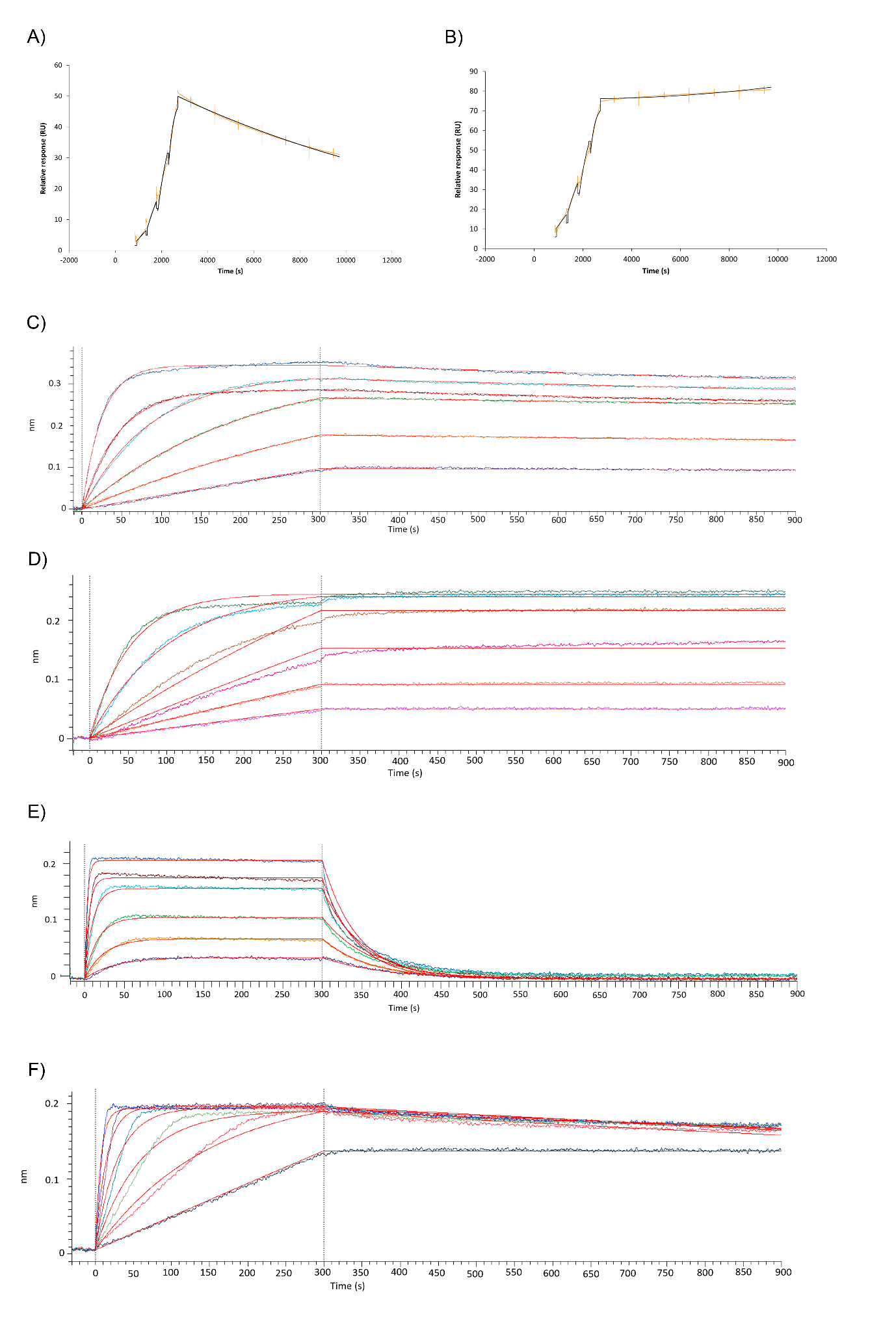


**Supplementary Figure 3. Binding sensorgrams and kinetic fitting curves obtained from surface plasmon resonance (SPR) and bio-layer interferometry (BLI). Panels A and B)** Binding curves from SPR (using single-cycle kinetics) illustrating the interaction between GTD and BL5.1 (A) and BL5.2 (B). **Panels C and D)** Sensorgrams from BLI showing the binding interaction between GTD and BL6.1 (C) and BL6.2 (D). **Panels** **E and F)** Binding curves from BLI depicting the interaction between GTD and BL7.1 (E) and BL7.2 (F).

**Table I**. **TcdB variants derived from different *C. difficile* strains.**

| **TcdB variant** | **Toxinotype** | ***C. difficile* strain** | **Ribotype** | **GTD identity (%)** |
| --- | --- | --- | --- | --- |
| 1 | 0 | VPI 10463 | 003/087 | 100 |
| 2 | III | R12087 (=CD196) | 027 | 96.5 |
| 3 | VIII | M68 | 017 | 78.6 |
| 4 | Xa | 036 / 591(CE) | 8864 | 78.8 |
| 5 | V | M120 | 078 | 98.1 |
| 6 | IV | CD04 | 023 | 98.3 |
| 7 | IXa | 51680 | 019 | 79 |
| 8 | XXX | ES130 | SLO 101 | 79.9 |
| 9 | IIa | SE 844 | 080 | 96.6 |

Classification of the toxinotypes derived of different *C. difficile* strains based on (Mansfield et al. 2020). Identity was calculated based on the amino acid sequence of the glucosyltransferase domain (GTD) of each TcdB toxinotype. GTD1 (Uniprot: P18177) served as the reference sequence for comparisons, with analyses conducted using the Omega clustering algorithm using CLC workbench software (Qiagen, Germany).
